# Copepod-Associated Gammaproteobacteria Respire Nitrate in the Open Ocean Surface Layers

**DOI:** 10.3389/fmicb.2018.02390

**Published:** 2018-10-10

**Authors:** Pia H. Moisander, Katyanne M. Shoemaker, Meaghan C. Daley, Elizabeth McCliment, Jennifer Larkum, Mark A. Altabet

**Affiliations:** ^1^Department of Biology, University of Massachusetts Dartmouth, North Dartmouth, MA, United States; ^2^School of Marine Science and Technology, University of Massachusetts Dartmouth, New Bedford, MA, United States

**Keywords:** denitrification, mesozooplankton, microbiome, nitrate, nitrogen budget, oxygen deficient zone

## Abstract

Microbial dissimilatory nitrate reduction to nitrite, or nitrate respiration, was detected in association with copepods in the oxygenated water column of the North Atlantic subtropical waters. These unexpected rates correspond to up to 0.09 nmol N copepod^−1^ d^−1^ and demonstrate a previously unaccounted nitrogen transformation in the oceanic pelagic surface layers. Genes and transcripts for both the periplasmic and membrane associated dissimilatory nitrate reduction pathways (Nap and Nar, respectively) were detected. The *napA* genes and transcripts were closely related with sequences from several clades of *Vibrio* sp., while the closest relatives of the *narG* sequences were *Pseudoalteromonas* spp. and *Alteromonas* spp., many of them representing clades only distantly related to previously described cultivated bacteria. The discovered activity demonstrates a novel Gammaproteobacterial respiratory role in copepod association, presumably providing energy for these facultatively anaerobic bacteria, while supporting a reductive path of nitrogen in the oxygenated water column of the open ocean.

## Introduction

Nitrogen (N) is the major nutrient limiting phytoplankton growth in vast regions of the oceans, including the North Atlantic Subtropical Gyre (NASG) (Moore et al., 2013) and its availability can control the efficiency of oceanic primary producers for fixing atmospheric CO_2_ (Karl et al., 1997). The recycling of organic N in the surface layers is primarily thought to occur via ammonification by heterotrophic bacteria, followed by rapid re-assimilation, or nitrification to nitrite (NO_2_^−^) and nitrate (NO_3_^−^) (Zehr and Kudela, 2011). In addition, a portion of organic N in particulate material sediments out of the euphotic layers and must be replaced by new supplies of fixed N. Thermodynamics predict that heterotrophic respiration by microbes in the oxygenated water columns generally rely on oxygen as the terminal electron acceptor. However, in marine anoxic and hypoxic oxygen deficient zones (ODZs) and sediments, NO_3_^−^ respiration is favored, leading to loss of fixed N to gaseous forms via the denitrification and anammox pathways (Devol, 2015). Where present, dissimilatory NO_3_^−^ reduction to ammonium (DNRA) can compete with NO_3_^−^ reduction for the NO_3_^−^ substrate (Smith et al., 2007; Lam and Kuypers, 2011; Stief et al., 2014; Yu et al., 2014; Devol, 2015).

Low oxygen microzones and associated microscale gradients in nutrients and gases can exist within live and dead particles and are potentially important sites of elemental fluxes in oceanic surface layers (Klawonn et al., 2015). As compared to free-living populations, distinct microbial communities are associated with particles and are largely thought to be driven by the availability of labile carbon and nutrient sources (Dang and Lovell, 2016). Live phytoplankton and zooplankton, including copepods, also host diverse bacterial communities (Carman and Dobbs, 1997; Hewson et al., 2009; Tang et al., 2010). Copepods, in particular, are abundant members of the zooplankton that are important for the trophic transfer of carbon, nitrogen, and other elements in marine food webs (Steinberg et al., 2002, 2008; Pendleton et al., 2009). These small crustaceans conduct daily vertical migrations across physical and chemical gradients in the water column and also produce microscale nutrient gradients (Frangoulis et al., 2005) that can attract marine chemotactic bacteria (Smriga et al., 2016). Bacterial associates are both attached to the copepod exoskeleton and found internally (Shoemaker and Moisander, 2017), such that the levels of oxygen they experience are likely to vary greatly. The intestinal tract of marine copepods can be anoxic (Tang et al., 2011), and associated bacteria include both facultative and obligate anaerobes, suggesting stable associations are present in this low oxygen microzone (Sochard et al., 1979; De Corte et al., 2014; Shoemaker and Moisander, 2015; Shoemaker and Moisander, 2017). Since NO_3_^−^ has a relatively high redox potential, we hypothesized that the prevailing availability of NO_3_^−^ and low O_2_ conditions within copepods allow associated bacteria to conduct some or all of the steps in the denitrification pathway for energy production. Such N removal from the bioavailable pool occurring at microscales in these highly abundant mesozooplankton is previously unknown but could have quantitative significance for the global marine N cycle.

Two major dissimilatory NO_3_^−^ reductase systems are known in bacteria, the *narGHI* (Nar) and *napABC* (Nap) pathways termed the membrane bound and periplasmic NO_3_^−^ reductases, respectively, and a third pathway, the *narXYV* system, is found in some organisms (Kraft et al., 2011). The Nar system, also referred to as the respiratory nitrate reductase, is found in diverse lineages, including at least Alpha-, Beta-, Gamma-, and Deltaproteobacteria, Firmicutes, Actinobacteria, and Archaea (Philippot, 2005). NarG, responsible for NO_3_^−^ reduction in the Nar pathway, is located in the bacterial cytoplasm and complexed to NarH, which connects it to the inner cytoplasmic membrane via NarI. In addition, a NO_3_^−^ transporter is necessary for bringing the substrate to the cytoplasm. The reduction of NO_3_^−^ to NO_2_^−^ via Nar results in proton motive force, which can be used to create ATP (Moreno-Vivian et al., 1999). Functional Nar is generally considered to require anaerobic conditions (Kraft et al., 2011).

The alternate NO_3_^−^ reductase (Nap) is located in the periplasm and has been described in a wide range of Gram-negative Proteobacteria (Flanagan et al., 1999; Philippot, 2005). Nap is active in many bacteria when oxygen is present (Siddiqui et al., 1993; Berks et al., 1995; Ji et al., 2015), although in others it may function under anaerobic conditions (Huang et al., 2015). The periplasmic Nap complex is not directly linked to proton translocation, but could provide energy when coupled to a respiratory dehydrogenase (Vazquez-Torres and Baeumler, 2016). The Nap system could serve as an electron sink to dissipate accumulating reducing power (Moreno-Vivian et al., 1999), and some organisms use it to provide substrate to denitrification or DNRA (Kraft et al., 2014). The function and gene organization of Nap appears to be more variable among bacteria than the membrane bound Nar (Gonzales et al., 2006). To date, most of the work on Nar and Nap has been on organisms from conventional environments such as sediments, wetlands, and ODZs, where denitrification is expected to occur due to known oxygen depletion. The activity and relative roles of Nar and Nap pathways in bacteria associated with live marine copepods appear to be unknown to date. In this study, we quantified N transformation rates and characterized copepod-associated bacteria for presence and expression of bacterial genes involved in these pathways in the oligotrophic NASG waters.

## Materials and Methods

### Overview of Sampling and Experimental Design

Zooplankton net sampling and experiments were conducted onboard the *R/V* Atlantic Explorer in August 2013 and 2014 (**Table 1**, **Supplementary Table S1**, and **Supplementary Methods**). The sampling was conducted at or in the vicinity of the Bermuda Atlantic Time Series Station (BATS). The location of the deep chlorophyll maximum (DCM) was estimated from hydrocasts conducted using the BATS methodology (Knap et al., 1994) and measuring conductivity, temperature and depth (CTD), chlorophyll a fluorescence, and oxygen concentration. The DCM remained at approximately 60–125 m during the 2014 cruise (**Supplementary Figure S1**). Other data collected by the BATS time series were used as background data (from the BATS permanent data repository^1^).

**Table 1:** Summary of experiments conducted in 2014.

Exp #	Net sample collection date	Lat, Lon	# Net tows pooled	Experiment type	Copepod # and type per vial	Incubation duration (h)	Incubation temperature (°C)
1	Aug 19, 2014	31.678°N, −64.195°W	3	*NR*	15 Mixed	36 h	27–34
2	Aug 21, 2014	31.553°N, −63.654°W	2	*NR*	15 Mixed	36 h	27–44
3	Aug 22, 2014	31.667°N, −64.108°W	3	*NR*	15 *Undinula*	36 h	12–31
4	Aug 23, 2014	31.667°N, −64.650°W	3	*NR*	15 Mixed	36 h	12–24
5	Aug 20, 2014	31.510°N, −64.333°W	4	*All-N*	10 Mixed	36 h	27–44
6	Aug 22, 2014	31.667°N, −64.108°W	3	*All-N*	15 *Undinula*	36 h	12–31
7	Aug 24, 2014	28.501°N, −64.500°W	3	*All-N*	15 Mixed	24 h	12–24

*“Mixed” = different copepod species were included. Net tows that were pooled were conducted immediately one after another; each tow lasted approximately 1 h from deployment to recovery. The location is an average between the replicate tows. NR vs. All-N: see text for details about experimental additions.*

Net tows were conducted between early evening and early morning immediately after the CTD cast at the station. A 200-μm mesh zooplankton net was lowered to approximately 50–90 m (final depth was determined from pressure sensor connected to the net frame). The mesh walls of the cod-end were sealed to minimize damage to zooplankton and minimum wire speeds (7–10 m^−1^ min^−1^) were used. Local time of tows on deck was between 16:30 and 05:20. Upon retrieval, the sample was immediately diluted into seawater obtained from the flow-through seawater system of the ship. To obtain a sufficient number of copepods, two to four consecutively sampled net tows from the same evening/night were pooled (**Table 1**). The goal was to collect a sufficient number of copepods for all of the experimental replicates as quickly as possible, and it was beyond the scope of this study to attempt to calculate volumetric zooplankton densities at each site from these samplings. Other studies from the extensive BATS time series exist on mesozooplankton density estimates (Steinberg et al., 2012).

Experiments examining rates of microbial N transformations were conducted in which copepods were incubated under a range of conditions. To initiate the experiments, live copepods were picked from pooled net tows and rinsed two to three times with filtered seawater (0.2 μm) (FSW) from the same station, then separated into batches in multi-well plates. Live copepods were kept suspended in gentle aeration in bins before picking, and chilled with ice packs. Approximately 15 live copepods were placed in FSW (**Table 1**). Separate triplicate vials (in rare cases duplicate) for each treatment were prepared for rate measurements, and for preserving samples for RNA and DNA analyses.

In order to obtain a sufficient number of animals in the experiments, several copepod species were included in most experiments, and the same number of each copepod type was distributed to each experimental vial. Copepods were not separated by sex or developmental stage. A sufficient number of *Undinula vulgaris* individuals was recovered that allowed conducting full experiments with them alone (experiments 3 and 6; **Table 1**). Other identified copepod species that were included in experiments with mixed communities included *Pleuromamma* sp. and *Sapphirina* sp.

### Preliminary Sample Collection in 2013

Preliminary experiments were conducted in 2013, to explore the influence of several ^15^N substrates on activities and transcription of copepod-associated bacteria (**Supplementary Methods**). Results were used to focus efforts in 2014. To explore overall microbial community transcription, six samples were processed for metatranscriptomes from experimental and net samples collected in 2013. Additionally, a few samples from the 2013 set were processed for amplification of *narG* and *napA* from DNA and cDNA (**Supplementary Table S1**). As the substrates were ^15^N labeled, the 2013 experiments provided initial preliminary data for the potential of NO_3_^−^ reduction in copepod associations (rate data not shown).

### Design of NR and All-N Experiments in 2014

All of the rate data and a majority of the molecular data shown are from experiments conducted in 2014. The experiments in 2014 included *NR experiments* that investigated nitrate reduction to nitrite (experiments 1–4) and separate *All-N experiments* (experiments 5–7) that investigated nitrite transformation to N_2_, NO_3_^−^, and NH_4_^+^ (**Table 1**). The experiments included copepod treatments with and without ^15^N tracer additions, and several copepod and seawater controls (see below). The ^15^N substrate additions are referred to as “tracers” below.

The *NR experiments* were conducted in 125-mL acid washed polycarbonate bottles, with 75 mL FSW amended with copepods, bacterial inoculum (5 mL unfiltered seawater from the site), nutrients, or tracers, in various combinations (see below). ^15^N-NO_3_^−^ was added (0.5 μM final), and ^14^N-NO_2_^−^ and ^14^N-NH_4_^+^ were added as “traps” (0.5 μM final), to assure sufficient background concentration for detection and increased turnover time of labeled product in the case of rapid loss of the product to other processes. The incubation (36 h in the dark) was terminated by filtration through combusted GF/F filters and the filtrate was saved and frozen for δ15N-DIN analysis (**Supplementary Methods**). The *All-N experiments* were conducted in gas tight, acid-washed 75-mL serum vials. To these experiments, ^15^N-NO_2_^−^ was added with ^14^N-NO_3_^−^ and ^14^N-NH_4_^+^ (all at 0.5 μM final concentration). The same copepod and seawater treatments were prepared as for *NR experiments*. The serum vials used in *All-N* experiments were filled with FSW after preparing the treatments, then sealed without bubbles using acid washed teflon-lined stoppers (Kimble Chase) and aluminum crimp seals. After the incubation, the activity in *All-N* vials used for measurements of different N products was terminated by adding mercuric chloride (100 μM final concentration) and vials were transported at ambient temperature to UMass Dartmouth for determination of different products (**Supplementary Methods**). Vials used for DNA and RNA analyses were prepared separately and collected at the beginning and at the end of the experiments.

Parallel treatments of seawater amended with a bacterial inoculum and nutrients served as controls to assess if free-living bacteria had the potential to carry out the same N transformations as those associated with copepods. These “bacterial inoculum” + nutrients treatments included phosphate (1 μM), nitrate (0.5 μM), ammonium (0.5 μM), iron (0.5 μM), EDTA (0.5 μM), and dextrose (0.5 μM). All vials were incubated in the dark on deck incubators with circulating surface water, in the lab at the ambient room temperature, or chilled with ice packs in coolers, for 24–36 h.

The treatments for both *NR* and *All-N* experiments included (1) 0.2-μm filtered seawater (FSW) + 15 copepod individuals + tracers, (2) FSW + 3–5 mL of unfiltered seawater serving as a bacterial inoculum (B) + tracers, (3) FSW + B + tracers + nutrients, (4) FSW + copepods, (5) FSW + B, (6) FSW + B + nutrients. Separate triplicate vials with these treatments were prepared for ^15^N incubations, and for collection of DNA and RNA. Treatments 1–3 were designed to obtain rate measurements and to collect DNA and RNA. Treatments 4–6 were intended as controls to exclude the influence of tracer additions, and used for additional DNA analyses. The bacterial inoculum was taken from seawater collected with Niskin bottles.

The potential products (^15^NO_2_^−^, ^15^NO_3_^−^, ^15^NH_4_^+^, and ^15^N_2_) from tracer addition treatments were detected using Isotope Ratio Mass Spectrometry (IRMS), and dissolved NO_2_^−^, NO_3_^−^, and NH_4_^+^ concentrations were measured colorimetrically (**Supplementary Methods**). For *All-N* experiments, O_2_ was measured by IRMS during the ^15^N_2_ analysis and quantified based on peak height and volume pumped to determine μM O_2_ present.

In the 2013 experiments, DNA and RNA were collected at the end of the experiment by filtering directly on the 0.2-μm filters without a pre-filter. In 2014, the samples were size fractionated by first filtering onto 10 μm polycarbonate filters (copepod size fraction), then 0.2 μm membrane filters (0.2–10 μm size fraction) (Supor, Pall Gelman) using 50-mL syringes, with two filter holders stacked. The filters were placed in sterile bead beater tubes with ∼0.1 g of 0.1 and 0.5-mm sterile glass beads, then immediately frozen in liquid N (RNA) or placed at −80°C (DNA). RNA tubes contained 350 μL RLT buffer with β-mercaptoethanol (Qiagen RNeasy kit). The T0 copepod samples were collected by adding copepods to experimental vials, which were then immediately filtered and preserved. Additional copepod samples were collected and preserved directly from the net tows for DNA and RNA analyses. These freshly collected live copepods were first rinsed with FSW several times, then picked into bead beater tubes and preserved as described above for RNA and DNA (**Supplementary Table S1**). All DNA and RNA samples (water samples on filters and copepods) were transported to UMass Dartmouth in a liquid nitrogen dry shipper.

### DNA and RNA Sample Collection and Processing

DNA was extracted from net copepod samples (picked copepod individuals or pooled copepod samples) and 0.2-μm and 10-μm filters (experiments), using a modified Qiagen DNeasy Plant Mini kit (Valencia, CA, United States) protocol (Moisander et al., 2008; Shoemaker and Moisander, 2015) (**Supplementary Methods** and **Supplementary Table S1**). RNA from parallel samples was extracted using the Qiagen RNeasy minikit (Qiagen) (**Supplementary Methods**). RNA (8 μL extract per reaction, maximizing the amount of RNA per reaction) was reverse transcribed to complementary DNA (cDNA) using the Superscript III kit (Thermo Fisher) and gene specific primers in separate reactions for each gene. Controls with no template or omitting the RT step were included and checked with PCR.

Metatranscriptome libraries were prepared to screen microbial genes transcribed in bacteria-copepod associations using a total of six samples (copepod net samples and experiments in 2013, **Supplementary Table S1**). The RNA used in metatranscriptome libraries was extracted as above then processed through additional steps to remove ribosomal RNA, to synthesize first and second strand cDNA, and to prepare the libraries for sequencing (**Supplementary Methods**). The libraries were sequenced at Tufts University using MiSeq (PE250). The data were trimmed, paired, and assembled in CLC Genomics Workbench 7 (CLC bio, Aarhus, Denmark), and analyzed using the MG-RAST metagenomics analysis server for metatranscriptome annotation (Meyer et al., 2008) (**Supplementary Methods**). The metatranscriptomes have the SRA accession number SRP089826. The *narG* and *napA* genes were separately amplified by PCR (DNA samples) and RT-PCR (cDNA generated from RNA), using previously published nested primers for *narG* (Gregory et al., 2000) and *napA* (Flanagan et al., 1999) (**Supplementary Methods**). The GenBank accession numbers for this study are MH586847–MH586927 for *napA* and MH586928–MH587013 for *narG*. The PCR and RT-PCR sequences were trimmed and conceptually translated in Arb (Ludwig et al., 2004). Neighbor-joining trees were constructed in Mega 6.06 (Tamura et al., 2013), using bootstrapping with 1,000 iterations to assign confidence levels to the branch nodes. Visualizations of the trees were generated and trees annotated in iTol (Letunic and Bork, 2007).

## Results

Hydrographic profiles from the Bermuda Atlantic Time Series Data show strong seasonal stratification with temperatures between 18 and 29°C in the surface 6–300 m layers, DCM at 60–125 m, and high ambient oxygen throughout the surface layers (**Supplementary Figure S1**). Dissolved nutrients were undetectable at the surface, and began increasing below approximately 125 m (**Supplementary Figure S2**). Nitrate reduction and the presence of genes and transcripts for both Nap and Nar pathways were detected in several experimental treatments containing copepods.

**FIGURE 1:**
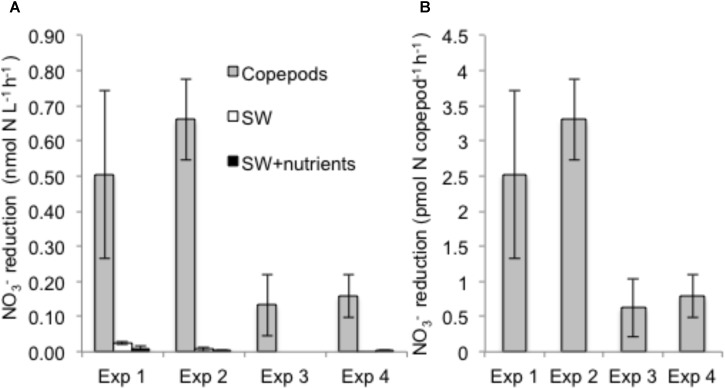
Nitrate reduction rates based on ^15^NO_2_^−^ production from added ^15^NO_3_^−^ tracer during four independent experiments. **(A)** Volumetric rates (nmol N L^−1^ h^−1^) as a function of treatment (“Copepods” – copepods + filtered seawater + tracers; “SW” – filtered seawater + whole seawater inoculum + tracers; “SW + nutrients” – filtered seawater + whole seawater inoculum + nutrients + tracers; **(B)** NO_3_^−^ reduction rate per copepod for the copepod treatments. Error bars indicate mean ± SD of three experimental replicates.

**FIGURE 2:**
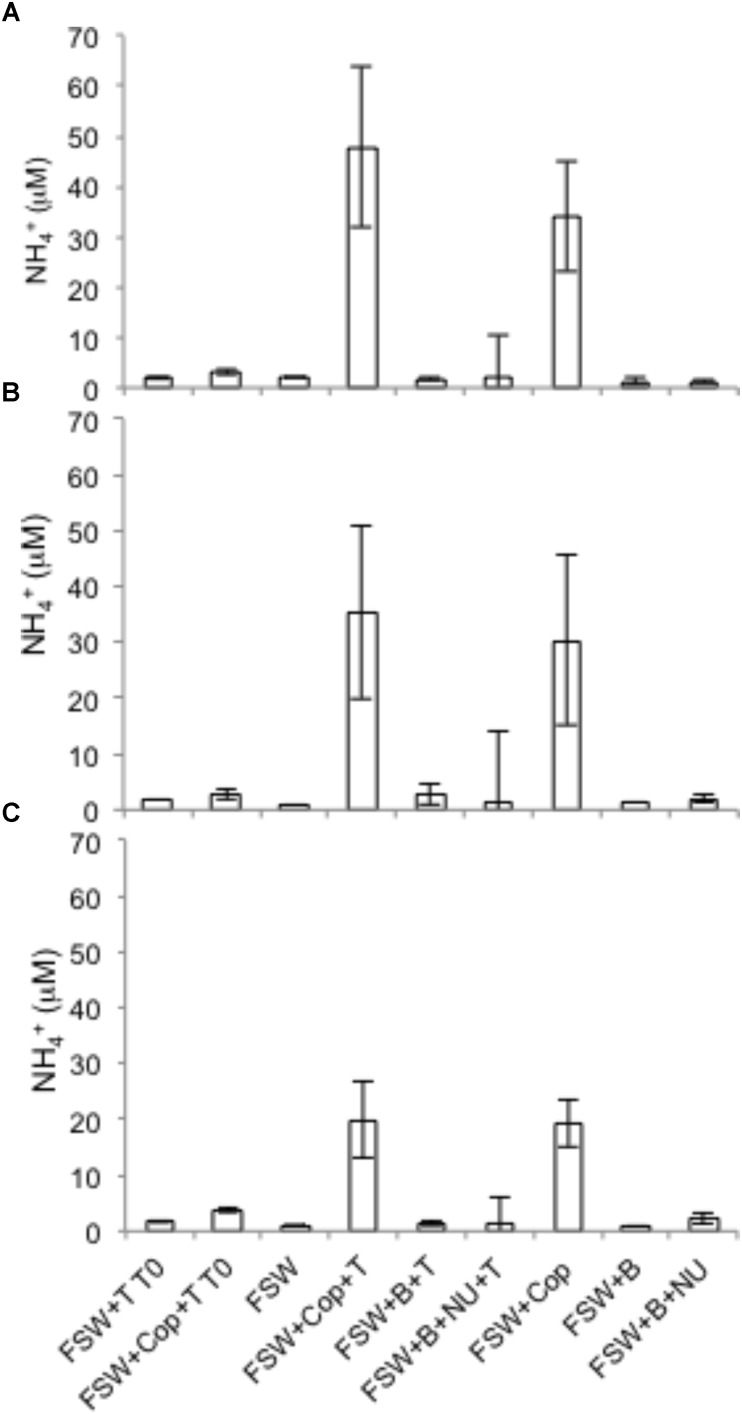
Total ammonium generation from copepods at the beginning (T0) or end of the incubation (36 h) in three NR experiments in 2014. FSW – 0.2 μm-filtered seawater; B – bacterial inoculum via addition of unfiltered seawater; Cop – copepods; T – tracers; NU – nutrients. **(A)** Experiment 1; **(B)** Experiment 3; **(C)** Experiment 4. T0 treatments were vials filtered immediately after addition of tracers and nutrient traps. The error bars indicate mean ± SD for three replicate experimental vials per treatment.

Nitrate reduction in copepods was evidenced by production of ^15^NO_2_^−^ when the substrate ^15^NO_3_^−^ was added, but rates were negligible in seawater controls with or without nutrient amendments (**Figure 1**). Rates of NO_3_^−^ reduction in copepod treatments were measured in all *NR* experiments (see “Materials and Methods”), conducted on different days and with different batches of copepods. NO_3_^−^ reduction rates varied from 0.07 to 0.78 nmol N L^−1^ h^−1^ (at a density of 15 copepods per vial) or 0.33 to 3.89 pmol N copepod^−1^ h^−1^ (up to 0.09 nmol N copepod^−1^ d^−1^) (**Figure 1**). Nitrate reduction was at or below the detection limit (0.03 nmol L^−1^ h^−1^) in the seawater treatments without copepods even if excess NO_3_^−^ or other nutrients and a carbon source were provided, indicating that free-living bacterioplankton in the surrounding seawater were not active. Production of ^15^N_2_ was not detected (*All-N* experiments) (detection limit 5 nmol L^−1^ h^−1^), suggesting the full denitrification pathway and anammox were not active under these experimental conditions. In *All-N* experiments, similar rates of NO_2_^−^ oxidation to NO_3_^−^ were detected in the copepod treatments (experimental averages 0.27–0.84 nmol L^−1^ h^−1^) and in seawater treatments without copepods (experimental averages 0.66–0.85 nmol L^−1^ h^−1^). The concentrations of inorganic nutrients in the *NR* experiments suggested a substantial net production of NH_4_^+^ from copepods (**Figure 2**), but production of ^15^NH_4_^+^ was not detected from ^15^NO_2_^−^ substrate additions. Oxygen was still present in the vials at the end of the incubations (**Supplementary Table S2**). The highest potential for O_2_ depletion would be expected to be present in the gas-tight serum vials (*All-N* experiments; see “Materials and Methods”). Whereas initial levels remained in the seawater incubations, O_2_ in the copepod incubations decreased to values as low as 14 mg O_2_ L^−1^. The NO_3_^−^ reduction measurements (*NR* experiments) were conducted in polycarbonate vials with a large air phase and non-gas-tight lids, thus it can be concluded that incubation vials in *NR* experiments were also oxygenated during the experiments.

**FIGURE 3:**
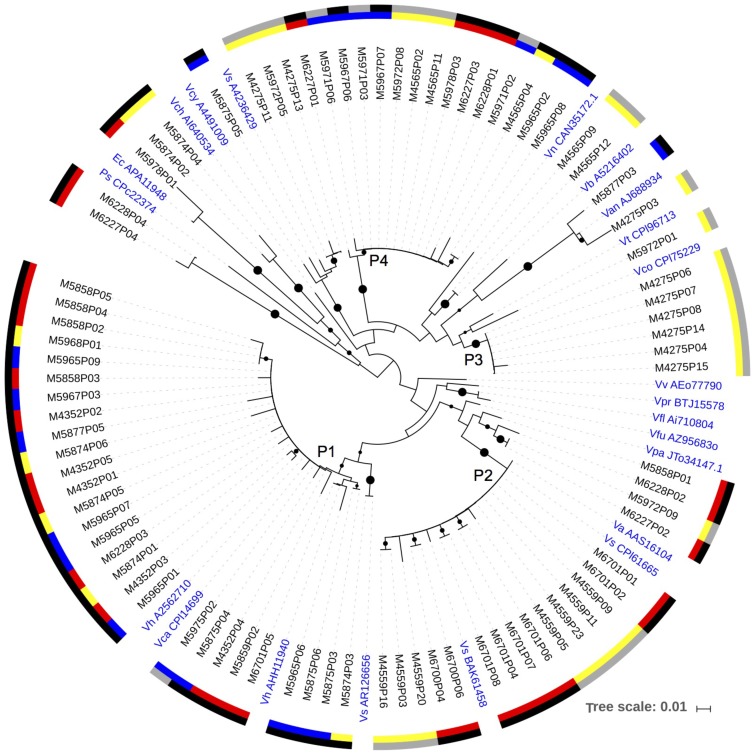
A neighbor-joining phylogenetic tree with the *napA* sequences obtained from the DNA and RNA samples. Bootstrap values over 50% are shown as black circles on the branches, based on 1,000 repeats, circle size indicating relative bootstrap value. The inner color scale: red – copepods obtained directly from the net samples; blue – 0.2–10 μm size fraction samples from experiments; yellow – size fraction containing copepods from experiments (>10 μm in 2014, and >0.2 μm in 2013). Outer scale: black – DNA; gray – RNA. Reference strains in blue: Vh – *Vibrio harveyi*; Vca – *Vibrio campbellii*; Vs – *Vibrio* sp.; Va – *Vibrio alginolyticus*; Vpa – *Vibrio parahaemolyticus*; Vfu – *Vibrio furnissii*; Vfl – *Vibrio fluvialis*; Vv – *Vibrio vulnificus*; Vco – *Vibrio corallilyticus*; Vt – *Vibrio tubiashii*; Van – *Vibrio angustum*; Vb – *Vibrio brasiliensis*; Vn – *Vibrio nigripulchritudo*; Vcy – *Vibrio cyclitrophicus*; Vch – *Vibrio cholerae*; Ec – *Escherichia coli*; Ps – *Pseudovibrio* sp.

DNA sequences and transcripts of *napA* were recovered from copepods collected directly from the net tows, copepods incubated in the experiments, and the 0.2–10 μm size fraction from the copepod incubations (**Figure 3**). A major cluster of *napA* sequences (Cluster P1) had a high amino acid identity to *Vibrio harveyi* (97–100% identity) and *V. campbellii* (97–99% identity). This clade included sequences from DNA samples extracted from individual copepods picked directly from the net tows such as *Undinula*, *Pleuromamma*, *Sapphirina*; DNA samples of both >10 (copepod size fraction) and 0.2–10 μm size fractions collected at the termination of experiments 1 and 2 in 2014; and an RNA sample from experiment 2. Another *napA* clade (P2) retrieved consistently had a 98–100% amino acid identity with *V. alginolyticus* (**Figure 3**). Cluster P2 contained both DNA and RNA sequences retrieved from both net tows and experiments. A third *napA* cluster (P3) consisting of only RNA sequences, had a relatively high identity with *V. corallilyticus*. A fourth major cluster of *napA* sequences (P4) had the highest identity (up to 100%) with *V. fortis* (NCBI reference WP_032552660.01). This cluster contained sequences from copepods collected from the net (*Sapphirina*, *Pseudocalanus*-like), from RNA in samples of the 0.2–10 μm and >10 μm size fractions of experiments, and DNA samples from the 0.2–10 μm size fraction of the experiments. In addition, a few of the *napA* sequences fell with other Gammaproteobacteria and away from *Vibrio* spp. (**Figure 3**). All *napA* sequences were relatively distant from the known pathogens *V. cholera, V. parahaemolyticus*, and *V. vulnificus* (**Figure 3**).

**FIGURE 4:**
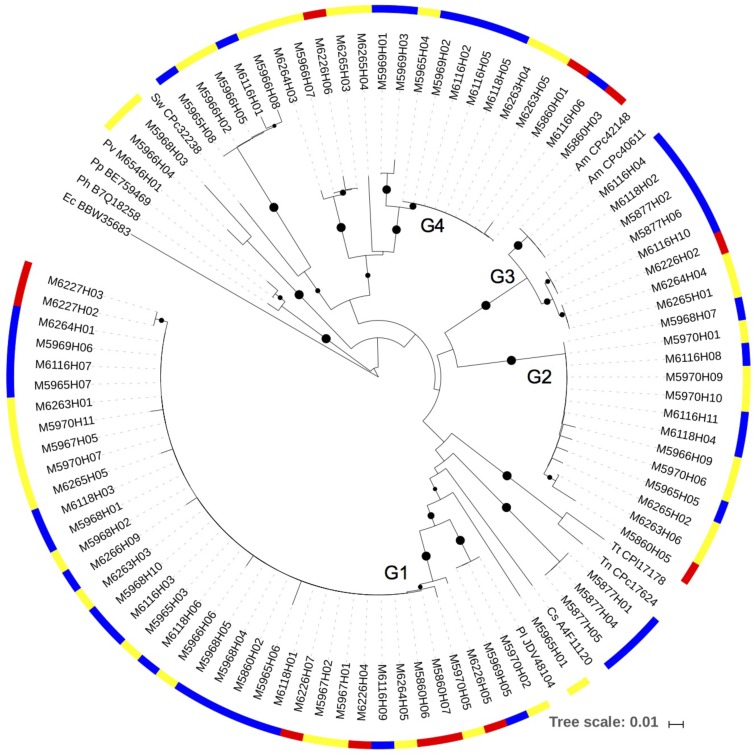
A neighbor-joining phylogenetic tree showing the *narG* sequences obtained from the DNA samples. Bootstrap values over 50% are shown as black circles on the branches, based on 1,000 repeats, circle size indicating relative bootstrap value. The color scale on the outer circle: red, copepods obtained directly from the net samples; blue, 0.2–10 μm size fraction samples from experiments; yellow, size fraction containing copepods from experiments (>10 μm in 2014, and >0.2 μm in 2013). Reference strains: Pl – *Pseudoalteromonas lipolytica*; Cs – *Cellvibrio* sp.; Tn – *Thioalkalivibrio nitratireducens*; Tt – *Thioalkalivibrio thiocyanoxidans*; Am – *Alteromonas macleodii*; Sw – *Shewanella woodyi*; Pv – *Proteus vulgaris*; Pp – *Proteus penneri*; Ph – *Proteus hauseri*; Ec –*Escherichia coli*.

The *narG* gene amplified consistently from copepod DNA samples collected directly from the net and from experiments (**Figure 4**). The sequences clustered into several clades, all within Gammaproteobacteria. The most commonly detected sequence was in the cluster with a closest match with *Pseudoalteromonas lipolytica* (90–100% amino acid identity) (Cluster G1). Sequences in this cluster were from DNA samples from copepods collected directly from the net, as well as samples collected at the end of experimental incubations. Many of the sequences also fell in the Cluster G2. The closest, although distant cultivated representative to G2 was the Gammaproteobacterium *Hahella ganghwensis* (NCBI reference WP_020407638.1) (with 88% amino acid identity). Another major clade (Cluster G3) had the best match to *Alteromonas macleodii* (97–100% amino acid identity). Blast searches with sequences from the fourth clade (Cluster G4) indicated there were no close cultivated relatives in GenBank. A small number of sequences fell away from these major clusters and had closer identities with other Gammaproteobacteria, including *Shewanella*.

Based on MG-RAST, 32–97% of the assembled metatranscriptome contigs were of bacterial origin (**Supplementary Table S3**). The metatranscriptomes contained transcripts from both Nap and Nar pathways. A total of 37 contigs from samples collected at the termination of experiments in 2013 contained *napA*; all of them mapped to different *Vibrio* spp. (**Supplementary Table S4**). Most of the *napA* sequences from the metatranscriptomes had the best match with *Vibrio* sp. Ex 25, which falls in the *V. campbellii/harveyi* cluster (*napA* Cluster P1; **Figure 3**), and other close matches included *V. splendidus*, *V. variabilis*, and *V. parahaemolyticus*. A few additional contigs from the metatranscriptomes, obtained from experiments and copepods collected directly from the net, were found that mapped to *narG* or *narY* (**Supplementary Table S5**), and *narK* (data not shown).

## Discussion

Dissimilatory NO_3_^−^ reduction to NO_2_^−^ was active in association with copepods in the oxygenated waters of the NASG, but the same activity was not detected in the surrounding seawater. This activity appears to be a previously unaccounted N transformation in oxygenated surface layers in the open ocean. NO_3_^−^ reduction rates in an ODZ off the Peruvian coast averaged 307 nmol L^−1^ d^−1^ (Lam et al., 2009) and up to 12.1 nmol L^−1^ d^−1^ in the Bay of Bengal ODZ (Bristow et al., 2017). Converted to daily rates, copepod associated activity detected in this study would be up to 0.09 nmol N copepod^−1^ d^−1^. If conservatively assuming the density of 1 copepod per L of Sargasso Sea seawater (Munk et al., 2010), copepod-based rates in these waters would be occurring at rates roughly two to three orders of magnitude lower than in these ODZs. Whereas ODZs are regionally limited, copepods are present throughout the oceanic surface and deep layers, thus such rates, when integrated to abundances of copepods in various marine environments, could be significant.

Both *napA* and *narG* genes and transcripts were detected, thus both of the processes are likely contributing to NO_3_^−^ reduction in the copepod microbiome. The higher abundance in the metatranscriptome data and consistent detection via RT-PCR suggest that the Nap pathway was more actively expressed than Nar. The Nap pathway is active under aerobic conditions in at least some bacteria (Berks et al., 1995), however, the Nar pathway appears to generally require anaerobic conditions; for example, in *Escherichia coli*, Nar is synthesized only under anaerobic conditions (Moreno-Vivian et al., 1999). Many sedimentary and soil systems appear to have both pathways present (Bru et al., 2007; Smith et al., 2007). The conditions in the copepod treatments were oxygenated, suggesting they were more likely to support activity in the Nap rather than Nar pathway, unless the process occurred in low O_2_ microzones internal to the copepods or was unconventional Nar activity under aerobic conditions (Qu et al., 2016; He et al., 2017; Medhi et al., 2017). Our previous data from BATS show presence of both *Vibrio* and *Pseudoalteromonas/Alteromonas* in the gut microbiomes of the copepods, but the relative abundances were greater when the entire copepod body was accounted for (Shoemaker and Moisander, 2015, 2017). These prior data suggest these bacteria localize both on the exoskeleton and gut of the copepods, and the latter may provide anoxia for transient or resident bacteria (Tang et al., 2011). In spite of reports from waste water and model systems, dissimilatory NO_3_^−^ reduction is generally not considered an active pathway in oxygenated surface waters of the ocean. However, recent measurements from marine sediments suggest NO_3_^−^ reduction activity is present under fluctuating oxygen conditions (Marchant et al., 2017). NarG is located in the cytoplasm, requiring a NO_3_^−^ transporter; thus access to the substrate could limit the rate of NO_3_^−^ reduction of the Nar pathway. The nitrate/nitrite transporter (*narK*) transcripts were detected in three of our metatranscriptome samples, as evidence for the activity. The periplasmic Nap does not require active transport and may have a lower affinity for NO_3_^−^ than found in NarG (Vazquez-Torres and Baeumler, 2016). The Nap pathway in fact appears to be more important than Nar for *E. coli* and *Salmonella enterica* under NO_3_^−^ deficiency (Stewart et al., 2002; Lopez et al., 2015). Better competitive fitness of organisms containing the Nap rather than the Nar pathway under low NO_3_^−^ was also suggested based on estuarine sediment bacterial *napA* and *narG* gene abundances under a NO_3_^−^ gradient (Dong et al., 2009). Overall, presence of the two alternate pathways with distinct oxygen dependencies and potentially different affinities or requirements for substrate should support active NO_3_^−^ reduction on copepod associations under a range of conditions.

The free-living bacterial community would be unlikely to have active NO_3_^−^ reduction in oligotrophic surface waters due to the requirement for NO_3_^−^, which is in short supply in these waters, as well as presence of high ambient oxygen (**Supplementary Figures S1, S2**). Lack of detectable rates in the seawater in this study suggested lack of such activity in the free-living communities. Further, supplying NO_3_^−^, nutrients, and carbon substrate to the free-living community did not stimulate NO_3_^−^ reduction rates in it within the time scale of the experiments, suggesting appropriate free-living communities were not readily present to initiate this activity.

Nitrate in the surface layers at BATS is below detection at this time of the year (**Supplementary Figure S2**), thus an alternate source for the substrate for NO_3_^−^ reduction would need to sustain the process. NO_3_^−^ reducing bacteria that are common in human oral and gut microbiomes are thought to obtain their substrate either directly from the host or host diet (Winter et al., 2013; Tiso and Schechter, 2015). Gut associated bacteria on marine crustaceans could also obtain their substrate either from sources originating from the host, or the bacteria could benefit from the copepod daily vertical migration (Steinberg et al., 2002; Sainmont et al., 2014) moving them to more NO_3_^−^ enriched deeper layers of the water column (Grossart et al., 2010). Variations in NO_3_^−^ reduction activity throughout the daily cycle could thus occur, related to changes in availability of substrate due to copepod feeding and migration. Copepods serve as a substantial source of NH_4_^+^ to their surroundings, as demonstrated by measurements here and elsewhere (Stock et al., 2014), and the data here also show that the presence of the NH_4_^+^ did not inhibit NO_3_^−^ reduction in copepod associations. Turbulent flow further away from the copepods is likely to quickly disperse the nutrient plumes surrounding them, but chemotaxis allows certain bacteria to take advantage of the variability in nutrients at small spatial scales (Smriga et al., 2016; Son et al., 2016). The NH_4_^+^ originating from copepods is most likely important in serving as a bacterial N source in these N limited surface waters, supporting bacterial assimilation, and could also provide substrate to the NO_3_^−^ reduction process, if a conversion path to NO_3_^−^ is present (typically, nitrification). Measured NO_2_^−^ oxidation rates demonstrated the presence of the second step of canonical nitrification in the incubations, resulting in production of NO_3_^−^ that could potentially support NO_3_^−^ reduction in microzones surrounding copepods.

The fate of the NO_2_^−^ formed from NO_3_^−^ reduction varies among bacteria. We did not detect ^15^N_2_ production in the copepod experiments, suggesting absence of the full denitrification and anammox pathways in the copepod microbiome. In contrast, these processes were reported in association with sinking detrital copepod carcass materials in an ODZ (Stief et al., 2017). The oxygenated conditions in our study may have prevented these processes, and active NO_2_^−^ oxidation at micro-oxic conditions also competes for the substrate (Bristow et al., 2017). If full denitrification is not present, Nap may also offer a mechanism for the cell to discard excess reductant (Berks et al., 1995), or NO_2_^−^ may be used in a competing process, DNRA (Kraft et al., 2014). However, lack of ^15^NH_4_^+^ production in copepod incubations confirms that the accumulating NH_4_^+^ originated from copepod waste products, not from DNRA. Active NO_2_^−^ oxidation would have removed at least some of the forming NO_2_^−^. It is possible that denitrification continued but stopped at NO or N_2_O, or NO_2_^−^ may have been removed by assimilation – these paths could not be deciphered with our measurements. The downstream steps of denitrification were not supported by metatranscriptomics, however.

Although the rates reported here demonstrate an active process, as in any incubation study, containment, including variability in temperature, may have influenced the relative level of activity. The two NO_3_^−^ reduction experiments conducted at cooler temperatures had the lowest NO_3_^−^ reduction rates (experiments 3 and 4), and lowest O_2_ utilization (experiment 7) supporting the idea that elevated temperatures enhanced the NO_3_^−^ reduction activity, along with the expected increase in copepod and microbial respiration. In their natural environment, vertically migrating copepods experience large variations in temperature (**Supplementary Figure S1**). The data presented here demonstrate the robustness of microbial processes under the relatively wide temperature range used in the incubation experiments.

The data from clone libraries and metatranscriptomics show that Nap and Nar pathways were used by different copepod-associated bacteria. While the primers used are degenerate, it is also possible the approach missed additional community members contributing to these processes. The independently obtained metatranscriptomic data support the conclusions from the PCR analyses. The Nap pathway on copepods was associated with a few dominant *Vibrio* spp., while the Nar pathway was primarily present in *Alteromonas* spp. and *Pseudoalteromonas* spp. related Gammaproteobacteria. *Vibrio* spp., have been identified as forming the majority of the copepod associated Gammaproteobacteria in the North Atlantic subtropical waters, determined by 16S rRNA amplicon sequencing (Shoemaker and Moisander, 2015). These facultative anaerobes should be able to benefit from the variable oxygen concentrations within and surrounding the copepods. They appear to form stable microbial communities on copepods (Colwell and Huq, 1999), and in the microenvironments provided by copepods, they are likely using distinct metabolic activities compared to their free-living counterparts. Marine particles are commonly colonized by the same general groups of Gammaproteobacteria as those detected in this study and should provide a comparison in future studies (Dang and Lovell, 2016).

The majority of *napA* sequences and transcripts had the closest matches with previously sequenced *Vibrio* spp. Searching with the protein family (pfam) 03892 for the Nap active site showed it is present in 1327 of the 1349 *Vibrio* spp. genomes (finished and draft genomes) in the Joint Genome Institute Integrated Microbial Genomes (JGI-IMG) database (September 2017). Nap is thus present in *Vibrio* spp. almost universally, suggesting it provides the genus key fitness benefits in their natural environments. In contrast, the Nar active site protein alpha chain (pfam 14710) was found in only 19 of the 1349 *Vibrio* spp. genomes, thus overall Nar is rare in *Vibrio* spp. The rest of the canonical denitrification steps are also rare in *Vibrio* spp. genomes; only 0–7 genomes contained nitrite, nitric oxide, and nitrous oxide reduction steps based on reported KEGG terms. Of the 166 *Alteromonas/Pseudoalteromonas* draft and finished genomes in JGI IMG, the Nap active site was found in only one *Pseudoalteromonas* genome (*P. denitrificans* DSM 6059), while the Nar alpha chain was found in five genomes. Thus neither the Nap nor Nar pathway appears to be common in *Pseudoalteromonas* spp. and *Alteromonas* spp. Sequences from this study were relatively close to these Gammaproteobacteria, but NO_3_^−^ respiring bacteria using either pathway within copepod associations appear to include many environmental lineages that have not been previously described from the perspective of these pathways. Evolutionary rates of 16S rRNA gene and *narG* appear different, which may complicate assignment of environmental *narG* sequences to species, however, horizontal gene transfer does not appear to be a strong forcing factor influencing either *narG* or *napA* gene distributions (Philippot, 2005; Palmer et al., 2009).

Active dissimilatory NO_3_^−^ reduction rates in association of abundant marine mesozooplankton in the oxygenated open ocean water column were unexpected. Although a few studies have reported low oxygen microzones in the oceanic water column beyond large scale ODZs, direct evidence and quantified estimates for dissimilatory nitrate reduction in these microzones is to our knowledge virtually non-existent. The results here suggest that while it may provide an energy source, NO_3_^−^ reduction within copepod guts or on their exoskeleton is not linked to production of N_2_. Attributing this activity to *Vibrio* and other abundant Gammaproteobacterial associates in the copepod microbiome shows an important biogeochemical process occurring via these associations. The results demonstrate a seldom-considered sink for NO_3_^−^ in the open ocean surface waters and suggest widespread presence of the initial step of denitrification in the zooplankton microhabitat surrounded by oxygenated oceanic water column. The focus of this study was to collect rate data and molecular evidence to demonstrate this active process. The complexity of the N cycle with numerous possible substrates and products makes tracing N in these dynamic microhabitats an exciting ongoing research topic.

## Data Availability Statement

The sequence datasets reported in this study can be found in the NCBI under SRP089826 and MH586847–MH587013.

## Author Contributions

PM and MA designed the study. PM, KS, MD, EM, and JL conducted the experiments. MA and JL analyzed the nutrients and stable isotope samples. PM, MD, and KS conducted the clone library analyses. EM collected the metatranscriptome data. KS and EM analyzed the data. PM wrote the paper with contributions from the other authors.

## Conflict of Interest Statement

The authors declare that the research was conducted in the absence of any commercial or financial relationships that could be construed as a potential conflict of interest.

## Footnotes

1 http://bats.bios.edu/data/
