# Supplementary material for: Copepod-Associated Gammaproteobacteria Respire Nitrate in the Open Ocean Surface Layers
Source: Front Microbiol. 2018 Oct 10;9:2390. doi: 10.3389/fmicb.2018.02390 (PMC6194322; doi:10.3389/fmicb.2018.02390)
Supplement: Supplementary file 1 [file Image_1.pdf]

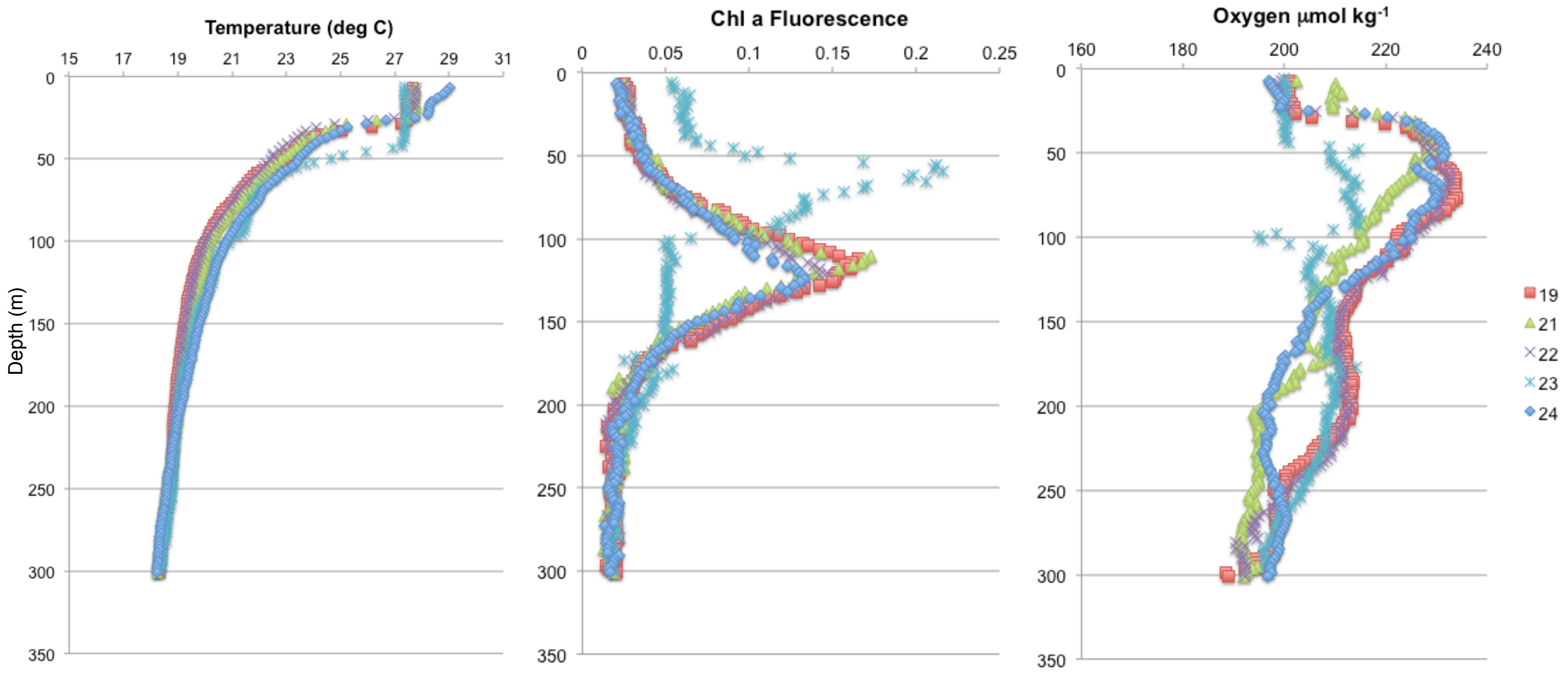

Figure S1. Temperature, chlorophyll *a* fluorescence, and oxygen profiles collected during the study in 19-24 August, 2014. The profiles were collected immediately before the first net tow at the station (see Table 1). The CTD was out of order on August 20th.
