# Supplementary figures and images for: Copepod-Associated Gammaproteobacteria Respire Nitrate in the Open Ocean Surface Layers
Source: Front Microbiol. 2018 Oct 10;9:2390. doi: 10.3389/fmicb.2018.02390 (PMC6194322; doi:10.3389/fmicb.2018.02390)

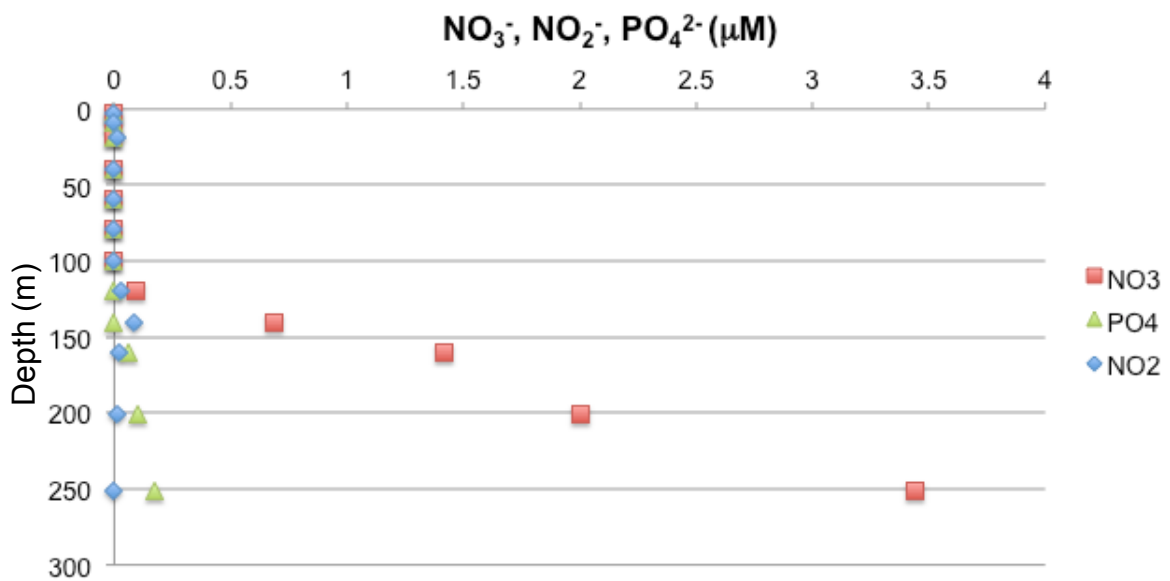

Figure S2. Nutrient profile at BATS in 22 August 2014 (<http://bats.bios.edu/data/>).

Supplement: Supplementary file 2 [file Image_2.pdf]
