# Supplementary material for: Copepod-Associated Gammaproteobacteria Respire Nitrate in the Open Ocean Surface Layers
Source: Front Microbiol. 2018 Oct 10;9:2390. doi: 10.3389/fmicb.2018.02390 (PMC6194322; doi:10.3389/fmicb.2018.02390)
Supplement: Supplementary file 3 [file Table_1.pdf]

Table S1. Samples included in the study. + amplification occurred in (RT-)PCR; - no amplification in (RT-)PCR; x - metatranscriptome analysis conducted; nd - not done. The >10 µm size fraction from experiments was considered the copepod size fraction. "Copepod" refers to copepods picked directly from net tow. Sample 6546 was a *Proteus vulgaris* culture used as a positive. PE - preliminary experiment; T0 - collected at the beginning of the experiment; Tend - collected at the end of the experiment.

**ID**	**Date**	**Time on deck**	**Station**	**Size fract. (mm)**	**Type**	**Exp # - time**	**Copepod type**	***napA***	***narG***	**Metatr.**
4275	8/2/13	5:18	BATS	>0.2	RNA	PE-T0	6 copepods	+	-	nd
4352	8/9/13	21:30	BATS	Copepod	DNA	Net	1 *Pleuromamma*	+	-	nd
4550	8/6/13	18:30	SS#13	Copepod	RNA	Net	5 *Undinula*	nd	nd	x
4559	8/6/13	18:30	SS#13	>0.2	RNA	PE-T0	11 copepods	+	-	nd
4565	8/7/13	18:50	E3	>0.2	RNA	PE-T0	12 copepods	+	-	nd
4569	8/8/13	13:25	SS#13	>0.2	RNA	PE-Tend	10 copepods	nd	nd	x
4570	8/8/13	13:25	SS#13	>0.2	RNA	PE-Tend	10 copepods	nd	nd	x
4646	8/9/13	21:05	E3	>0.2	RNA	PE-Tend	10 *Undinula*	nd	nd	x
4647	8/9/13	21:05	E3	>0.2	RNA	PE-Tend	10 *Undinula*	nd	nd	x
4648	8/9/13	21:05	E3	>0.2	RNA	PE-Tend	10 *Undinula*	nd	nd	x
5858	8/21/14	16:25	BATS	Copepod	DNA	Net	1 *Undinul*a	+	-	nd
5859	8/22/14	16:25	BATS	Copepod	DNA	Net	1 *Undinula*	+	nd	nd
5860	8/22/14	16:25	BATS	Copepod	DNA	Net	1 *Undinula*	nd	+	nd
5874	8/21/14	1:30	PITTS	>10	DNA	1-Tend	15 copepods	+	-	nd
5875	8/21/14	1:30	PITTS	0.2-10	DNA	1-Tend	15 copepods	+	-	nd
5877	8/21/14	1:30	PITTS	0.2-10	DNA	1-Tend	15 copepods	nd	+	nd
5965	8/23/14	1:05	SS#13	0.2-10	DNA	2-Tend	15 copepods	+	+	nd
5966	8/23/14	1:05	SS#13	>10	DNA	2-Tend	15 copepods	nd	+	nd
5967	8/23/14	1:05	SS#13	0.2-10	DNA	2-Tend	15 copepods	+	+	nd
5968	8/23/14	1:05	SS#13	>10	DNA	2-Tend	15 copepods	+	+	nd
5969	8/23/14	1:05	SS#13	0.2-10	DNA	2-Tend	15 copepods	nd	+	nd
5970	8/23/14	1:05	SS#13	>10	DNA	2-Tend	15 copepods	nd	+	nd

5971	8/23/14	1:05	SS#13	0.2-10	RNA	2-Tend	15 copepods	+	-	nd
5972	8/23/14	1:05	SS#13	>10	RNA	2-Tend	15 copepods	+	-	nd
5975	8/23/14	1:05	SS#13	0.2-10	RNA	2-Tend	15 copepods	+	nd	nd
5978	8/23/14	1:05	SS#13	Copepod	DNA	Net	15 copepods	+	-	nd
6116	8/24/14	18:00	BATS	0.2-10	DNA	3-Tend	15 *Undinula*	nd	+	nd
6118	8/24/14	18:00	BATS	0.2-10	DNA	3-Tend	15 *Undinula*	nd	+	nd
6226	8/25/14	21:30	AC1	Copepod	DNA	Net	1 *Undinula*	nd	+	nd
6227	8/25/14	21:30	AC1	Copepod	DNA	Net	1 *Sapphirina*	+	+	nd
6228	8/25/14	21:30	AC1	Copepod	DNA	Net	1 *Sapphirina*	+	nd	nd
6263	8/25/14	21:30	AC1	>10	DNA	4-Tend	15 copepods	nd	+	nd
6264	8/25/14	21:30	AC1	0.2-10	DNA	4-Tend	15 copepods	+	+	nd
6265	8/25/14	21:30	AC1	>10	DNA	4-Tend	15 copepods	nd	+	nd
6266	8/25/14	21:30	AC1	0.2-10	DNA	4-Tend	15 copepods	+	+	nd
6546			Positive ctr	*P. vulgaris*	DNA	n/a	n/a	nd	+	nd
6700	8/23/14	20:00	SS#1 -6	Copepod	DNA	Net	2 copepods	+	-	nd
6701	8/25/14	21:30	AC1	Copepod	DNA	Net	3 copepods	+	-	nd
