# Supplementary material for: Copepod-Associated Gammaproteobacteria Respire Nitrate in the Open Ocean Surface Layers
Source: Front Microbiol. 2018 Oct 10;9:2390. doi: 10.3389/fmicb.2018.02390 (PMC6194322; doi:10.3389/fmicb.2018.02390)
Supplement: Supplementary file 4 [file Table_2.pdf]

Table S2. Oxygen concentration in "*All-N*" experiments. Exp - experiment ID; FSW - filtered seawater; Cop - copepods; T - tracers; B - bacterial inoculant (unfiltered seawater added); NU - nutrients; T0 - time zero; incubated - measured at the end of the incubation.

**Exp**	**Treatment**	**Abbreviation**	**O_2 (µmol L^{-1})**
5	T0 FSW+tracers; killed at T0	FSW+T T0	167.66
5	T0 FSW+tracers; killed at T0	FSW+T T0	167.64
5	T0 FSW+Copepods+tracers; killed at T0	FSW+Cop+T T0	168.23
5	T0 FSW+Copepods+tracers; killed at T0	FSW+Cop+T T0	157.73
5	FSW+Copepods+tracers; incubated	FSW+Cop+T	36.09
5	FSW+Copepods+tracers; incubated	FSW+Cop+T	22.17
5	FSW+seawater inoculum+tracers; incubated	FSW+B+T	158.41
5	FSW+seawater inoculum+tracers; incubated	FSW+B+T	158.69
5	FSW+SW inoculum+nutrients+tracers; incubated	FSW+B+NU+T	160.88
5	FSW+SW inoculum+nutrients+tracers; incubated	FSW+B+NU+T	157.10
6	FSW+tracers; killed at T0	FSW+T T0	178.96
6	FSW+tracers; killed at T0	FSW+T T0	176.90
6	Copepods+tracers; killed at T0	FSW+Cop+T T0	170.25
6	Copepods+tracers; killed at T0	FSW+Cop+T T0	160.74
6	FSW+seawater inoculum+tracers; incubated	FSW+B+T	173.69
6	FSW+seawater inoculum+tracers; incubated	FSW+B+T	186.60
6	FSW+Copepods; incubated	FSW+Cop	17.22
6	FSW+Copepods; incubated	FSW+Cop	70.16
6	FSW+Copepods+tracers; incubated	FSW+Cop+T	14.10
6	FSW+Copepods+tracers; incubated	FSW+Cop+T	13.99
6	FSW+Copepods+tracers; incubated	FSW+Cop+T	45.31
6	FSW+SW inoculum+tracers; incubated	FSW+B+T	180.53
6	FSW+SW inoculum+tracers; incubated	FSW+B+T	173.05
6	FSW+SW inoculum+tracers; incubated	FSW+B+T	171.74
6	FSW+SW inoculum+nutrients+tracers; incubated	FSW+B+NU+T	170.82
6	FSW+SW inoculum+nutrients+tracers; incubated	FSW+B+NU+T	173.08
6	FSW+SW inoculum+nutrients+tracers; incubated	FSW+B+NU+T	173.67
7	FSW+tracer; killed at T0	FSW+T T0	168.36
7	FSW+tracer; killed at T0	FSW+T T0	176.38
7	FSW+Copepods+tracers; killed at T0	FSW+Cop+T T0	168.26
7	FSW+Copepods+tracers; killed at T0	FSW+Cop+T T0	169.25
7	FSW+Copepods; killed at T0	FSW+Cop T0	175.76
7	FSW+Copepods; killed at T0	FSW+Cop T0	176.33
7	FSW+seawater inoculum; incubated	FSW+B	132.43
7	FSW+seawater inoculum; incubated	FSW+B	121.71
7	FSW+copepods; incubated	FSW+Cop	113.54
7	FSW+copepods; incubated	FSW+Cop	141.29
