# Supplementary material for: Copepod-Associated Gammaproteobacteria Respire Nitrate in the Open Ocean Surface Layers
Source: Front Microbiol. 2018 Oct 10;9:2390. doi: 10.3389/fmicb.2018.02390 (PMC6194322; doi:10.3389/fmicb.2018.02390)
Supplement: Supplementary file 5 [file Table_3.pdf]

Table S3. General information about the metatranscriptomes collected in this study. # Contigs is the number of sequences after the reads were assembled. The balance of % sequences remaining after the sum of bacterial and eukaryotic are archaeal+viral (data from MG-RAST).

**Sample**	**Sample information**	**Date collected**	**# Contigs**	**Average length of contigs (bp)**	**% bacterial contigs**	**% eukaryotic contigs**	**% of all contigs assigned to nitrogen metabolism**
4550	5 *Undinula* individuals from net	6 Aug, 2013	8,812	225	31.95	67.53	0.44
4569	10 copepods from experiment	8 Aug, 2013	31,673	273	97.23	2.74	0.66
4570	10 copepods from experiment	8 Aug, 2013	33,894	279	96.95	2.99	0.90
4646	10 *Undinula* from experiment	9 Aug, 2013	22,809	285	86.45	13.52	0.59
4647	10 *Undinula* from experiment	9 Aug, 2013	11,432	244	74.85	25.13	0.70
4648	10 *Undinula* from experiment	9 Aug, 2013	20,316	267	83.22	16.76	0.69
