# Supplementary material for: Copepod-Associated Gammaproteobacteria Respire Nitrate in the Open Ocean Surface Layers
Source: Front Microbiol. 2018 Oct 10;9:2390. doi: 10.3389/fmicb.2018.02390 (PMC6194322; doi:10.3389/fmicb.2018.02390)
Supplement: Supplementary file 6 [file Table_4.pdf]

Table S4. Reads from the metatranscriptomes from all samples collected in 2013 that had reads mapping to *napA*, with the identity of the closest match shown as identified by MG-RAST. Sample identifiers: 4569, 4570 - Preliminary Experiment from station SS#13, end time point; 4646, 4647, 4648 - Preliminary Experiment from station E3, end time point.

>4637929.3|Sample_4569.R1_ contig_23858_1_207_+Vibrio sp. Ex25, 100% identity, napA
CCTATCGAAGAAGCAAATGAATTGAACGATGACGCTCATCACTTTGGCTATTACGTT
CAAAAAGGTCTATTTGAGGAATACGCTCAGTTCGGTCGCGGCCATGGCCACGATTTA
GCACCGTATGATCGTTACCACGAAGTACGCGGTTTACGTTGGCCGGTAGTAGATGGT
AAAGAAACGCTTTGGCGCTACAAAGAAGGCTCA

>4637934.3|Sample_4647.R1_ contig_5394_1_212_+Vibrio sp. Ex25, 100% identity, napA
TGTATGAACCAGCATACTCGTGGTGTATGGATGAATAACTTGGTTTACAACATCCAC
CTACTAACCGGTAAGATCTCGACTCCGGGGAACAGCCCATTCTCACTAACGGGCCAA
CCATCAGCATGTGGTACCGCTCGTGAGGTTGGTACGTTTGCTCACCGCCTACCAGCG
GACATGGTGGTTGCTAACCCTAAACACCGTAAGATTGCA

>4637933.3|Sample_4646.R1_ contig_14221_1_182_+Vibrio sp. Ex25, 98% identity, napA
TCTGGAACACAACATTCCCAAATCGAGGGTGAAGATGAAAATGACAAGAGTGCGTT
TGTGAAAGCAAACGCGGCTGCATCAGCTGCTGCTGTCGCAGGTGTGACACTACCAGC
ATCTGCAGCCAACCTGATTGCAAGCTCTGATCAAACCAAAATCACATGGGACAAAGC
ACTTGTC

>4637930.3|Sample_4570.R1_ contig_31537_1_205_+Vibrio sp. Ex25, 100% identity, napA
GTGGGCACAGTCGGTCAAGCAAAATCGGACCTTTGGCAACTAATGGAGTTCTCGAAA
CGCTTCAAAATGGAAGAAGTGTGGCCTGAAGAGCTTCTAGCGAAAGCGCCTCAATAC
CGTGGTAAAACAATGTACGACGTACTTTACCGCAATGGTAACGTCGACAAATTCCCT
ATCGAAGAAGCTCGTGAACTGAATGACGAT

>4637930.3|Sample_4570.R1_ contig_1326_44_246_+Vibrio sp. Ex25, 93% identity, napA
GCAGAGATGCACCCGGTTCTATGGACTCGCATTACTGACCGCCGCCTAAGCCACCCT
CACGTTAAAGTGAACGTGCTTTCTACTTACTACCATCGTTCATTTGAGTTGGCAGACC
ACGGCTACATTTTCAATCCTCAGTCTGACCTTGCAATTGCTAACTTCATCGCAAACTA
CATCATCGAAAACGATGCAGTAAAC

>4637930.3|Sample_4570.R1_ contig_7739_1_179_+Vibrio sp. Ex25, 100% identity, napA
TGTCTATTTGAGGAATACGCTCAGTTCGGTCGCGGCCATGGCCACGATTTAGCACCG
TATGATCGTTACCACGAAGTACGCGGTTTACGTTGGCCGGTAGTAGATGGTAAAGAA
ACGCTTTGGCGCTACAAAGAAGGCTCAGATCCCTATGCAAAAGCGGGTTCAGGTTGG
GACTTC

>4637929.3|Sample_4569.R1_ contig_28772_1_191_+Vibrio sp. Ex25, 93% identity, napA
CATGATTTGGCTCCATACGACCGCTACCACCAAGTACGTGGTCTACGTTGGCCTGTTG
TTGATGGTAAGGAAACACAGTGGCGCTTTAAAGAAGGTTCAGACCCATACGCGAAA
GAAGGTTCTGGTTGGGACTTCTACGGCAATGCTGACGGCAAAGCGAAGATCATTTCC
GCGCCATACGAAGCG

>4637933.3|Sample_4646.R1_ _contig_20262_1_189_+Vibrio sp. Ex25, 100% identity, napA
TACCGCCTACCAGCGGACATGGTGGTTGCTAACCCTAAACACCGTAAGATTGCAGAA
GACATCTGGAAACTGCCAGAAGGTACGATTCCACCAAAACCTGGCTTCCACGCCGTA

TTGCAAGACCGTATGCTGAATGACGGCGTTCTGAACTGTTACTGGGTTCAATGTAAC
AACAACATGCAAGCC

>4637930.3|Sample_4570.R1_ contig_31537_1_205_+Vibrio sp. Ex25, 100% identity, napA
GTGGGCACAGTCGGTCAAGCAAAATCGGACCTTTGGCAACTAATGGAGTTCTCGAAA
CGCTTCAAAATGGAAGAAGTGTGGCCTGAAGAGCTTCTAGCGAAAGCGCCTCAATAC
CGTGGTAAAACAATGTACGACGTACTTTACCGCAATGGTAACGTCGACAAATTCCCT
ATCGAAGAAGCTCGTGAACTGAATGACGAT

>4637929.3|Sample_4569.R1_ contig_28772_1_191_+Vibrio sp. Ex25, 100% identity, napA
CATGATTTGGCTCCATACGACCGCTACCACCAAGTACGTGGTCTACGTTGGCCTGTTG
TTGATGGTAAGGAAACACAGTGGCGCTTTAAAGAAGGTTCAGACCCATACGCGAAA
GAAGGTTCTGGTTGGGACTTCTACGGCAATGCTGACGGCAAAGCGAAGATCATTTCC
GCGCCATAGAAGCG

>4637933.3|Sample_4646.R1_ contig_20262_1_189_+Vibrio parahaemolyticus, 95% identity, napA
TACCGCCTACCAGCGGACATGGTGGTTGCTAACCCTAAACACCGTAAGATTGCAGAA
GACATCTGGAAACTGCCAGAAGGTACGATTCCACCAAAACCTGGCTTCCACGCCGTA
TTGCAAGACCGTATGCTGAATGACGGCGTTCTGAACTGTTACTGGGTTCAATGTAAC
AACAACATGCAAGCC

>4637929.3|Sample_4569.R1_ contig_23858_1_207_+Vibrio sp. Ex25, 100% identity, napA
CCTATCGAAGAAGCAAATGAATTGAACGATGACGCTCATCACTTTGGCTATTACGTT
CAAAAAGGTCTATTTGAGGAATACGCTCAGTTCGGTCGCGGCCATGGCCACGATTTA
GCACCGTATGATCGTTACCACGAAGTACGCGGTTTACGTTGGCCGGTAGTAGATGGT
AAAGAAACGCTTTGGCGCTACAAAGAAGGCTCA

>4637934.3|Sample_4647.R1_contig_5394_1_212_+Vibrio sp. Ex25, 93% identity, napA
TGTATGAACCAGCATACTCGTGGTGTATGGATGAATAACTTGGTTTACAACATCCAC
CTACTAACCGGTAAGATCTCGACTCCGGGGAACAGCCCATTCTCACTAACGGGCCAA
CCATCAGCATGTGGTACCGCTCGTGAGGTTGGTACGTTTGCTCACCGCCTACCAGCG
GACATGGTGGTTGCTAACCCTAAACACCGTAAGATTGCA

>4637933.3|Sample_4646.R1_contig_14221_1_182_+Vibrio parahaemolyticus, 100% identity, napA
TCTGGAACACAACATTCCCAAATCGAGGGTGAAGATGAAAATGACAAGAGTGCGTT
TGTGAAAGCAAACGCGGCTGCATCAGCTGCTGCTGTCGCAGGTGTGACACTACCAGC
ATCTGCAGCCAACCTGATTGCAAGCTCTGATCAAACCAAAATCACATGGGACAAAGC
ACTTGTC

>4637930.3|Sample_4570.R1_contig_14670_1_235_+Vibrio sp. Ex25, 93% identity, napA
TGGCTATGTACTGGCCGTGTGCTTGAACATTGGCACACCGGTACTATGACGCGACGC
GTACCTGAGTTGTATAAAGCGGTACCTGATGCTGTCTGCTACATGCACCCAGACGAT
GCGAAGAAACGTAACGTTCGTCGTGGTGAAGAAGTTCTGATCACAAACAAACGCGG
TGAAGTACGCGTTCGTGTAGAAACCCGCGGCCGTAACCGTCCACCACAAGGCTTAGT
GTTT

>4637930.3|Sample_4570.R1_contig_1326_44_246_+Vibrio sp. Ex25, 93% identity, napA
GCAGAGATGCACCCGGTTCTATGGACTCGCATTACTGACCGCCGCCTAAGCCACCCT
CACGTTAAAGTGAACGTGCTTTCTACTTACTACCATCGTTCATTTGAGTTGGCAGACC

ACGGCTACATTTTCAATCCTCAGTCTGACCTTGCAATTGCTAACTTCATCGCAAACTA
CATCATCGAAAACGATGCAGTAAAC

>4637930.3|Sample_4570.R1_ contig_7739_1_179_+Vibrio sp. Ex25, 100% identity, napA
TGTCTATTTGAGGAATACGCTCAGTTCGGTCGCGGCCATGGCCACGATTTAGCACCG
TATGATCGTTACCACGAAGTACGCGGTTTACGTTGGCCGGTAGTAGATGGTAAAGAA
ACGCTTTGGCGCTACAAAGAAGGCTCAGATCCCTATGCAAAAGCGGGTTCAGGTTGG
GACTTC

>4637935.3|Sample_4648.R1_contig_11818_1_339_+Vibrio variabilis, 93% identity, napA
CTGGCTAAAGCACCGCAATACCGCGGCAAAACCATGTACGACATGCTATTTGCTAAC
GGTAGCGTTGATAAATTCCCTATCGAAGAAGCAAATGAATTGAACGATGACGCTCAT
CACTTTGGCTATTACGTTCAAAAAGGTCTATTTGAGGAATACGCTCAGTTCGGTCGCG
GCCATGGCCACGATTTAGCACCGTATGATCGTTACCACGAAGTACGCGGTTTACGTT
GGCCGGTAGTAGATGGTAAAGAAACGCTTTGGCGCTACAAAGAAGGCTCAGATCCC
TATGCAAAAGCGGGTTCAGGTTGGGACTTCTACGGCAAGCCAGACGGCAAG

>4637933.3|Sample_4646.R1 _contig_13573_1_341_+Vibrio sp. Ex25, 100% identity, napA
GGTACGGGTTGTTCAGTACTGGTAGGTACTCAAAATGGTAAAGTGGTTGCGACTCAA
GGCGACCCAGAAGCACCAGTAAACAAAGGTCTTAACTGTATCAAAGGCTACTTCCTT
ACAAAAATCATGTACGGTAAGGATCGTCTAGATACGCCGCTACTTCGTATGAAAGAT
GGCAAATTCCATAAAGATGGTGATTTCGCACCAGTATCTTGGGATCAAGCTTTCGAC
GTAATGGCTGAAAAGTGGAAAGAAGCGCTGGCTAAGAAAGGTCCAACATCTGTTGG
TATGTTCGGCTCTGGTCAATGGACAGTAATGGAAGGTTATGCTGCTGCAAAAATG

>4637929.3|Sample_4569.R1_contig_28772_1_191_+Vibrio sp. Ex25, 98% identity, napA
CATGATTTGGCTCCATACGACCGCTACCACCAAGTACGTGGTCTACGTTGGCCTGTTG
TTGATGGTAAGGAAACACAGTGGCGCTTTAAAGAAGGTTCAGACCCATACGCGAAA
GAAGGTTCTGGTTGGGACTTCTACGGCAATGCTGACGGCAAAGCGAAGATCATTTCC
GCGCCATACGAAGCG

>4637933.3|Sample_4646.R1_ contig_14221_1_182_+Vibrio sp. Ex25, 93% identity, napA
TCTGGAACACAACATTCCCAAATCGAGGGTGAAGATGAAAATGACAAGAGTGCGTT
TGTGAAAGCAAACGCGGCTGCATCAGCTGCTGCTGTCGCAGGTGTGACACTACCAGC
ATCTGCAGCCAACCTGATTGCAAGCTCTGATCAAACCAAAATCACATGGGACAAAGC
ACTTGTC

>4637929.3|Sample_4569.R1_contig_23858_1_207_+Vibrio splendidus, 91% identity, nitrate reductase catalytic subunit PRK
CCTATCGAAGAAGCAAATGAATTGAACGATGACGCTCATCACTTTGGCTATTACGTT
CAAAAAGGTCTATTTGAGGAATACGCTCAGTTCGGTCGCGGCCATGGCCACGATTTA
GCACCGTATGATCGTTACCACGAAGTACGCGGTTTACGTTGGCCGGTAGTAGATGGT
AAAGAAACGCTTTGGCGCTACAAAGAAGGCTCA

>4637930.3|Sample_4570.R1_contig_7739_1_179_+Vibrio sp. Ex25, 100% identity, napA
TGTCTATTTGAGGAATACGCTCAGTTCGGTCGCGGCCATGGCCACGATTTAGCACCG
TATGATCGTTACCACGAAGTACGCGGTTTACGTTGGCCGGTAGTAGATGGTAAAGAA
ACGCTTTGGCGCTACAAAGAAGGCTCAGATCCCTATGCAAAAGCGGGTTCAGGTTGG
GACTTC

>4637930.3|Sample_4570.R1_contig_31537_1_205_+Vibrio splendidus LGP32, 90% identity, nitrate reductase catalytic subunit PRK
GTGGGCACAGTCGGTCAAGCAAAATCGGACCTTTGGCAACTAATGGAGTTCTCGAAA
CGCTTCAAAATGGAAGAAGTGTGGCCTGAAGAGCTTCTAGCGAAAGCGCCTCAATAC
CGTGGTAAAACAATGTACGACGTACTTTACCGCAATGGTAACGTCGACAAATTCCCT
ATCGAAGAAGCTCGTGAACTGAATGACGAT

>4637934.3|Sample_4647.R1_contig_5394_1_212_+Vibrio sp. Ex25, 100% identity, napA
TGTATGAACCAGCATACTCGTGGTGTATGGATGAATAACTTGGTTTACAACATCCAC
CTACTAACCGGTAAGATCTCGACTCCGGGGAACAGCCCATTCTCACTAACGGGCCAA
CCATCAGCATGTGGTACCGCTCGTGAGGTTGGTACGTTTGCTCACCGCCTACCAGCG
GACATGGTGGTTGCTAACCCTAAACACCGTAAGATTGCA

>4637930.3|Sample_4570.R1_contig_1326_44_246_+Vibrio sp. Ex25, 100% identity, napA
GCAGAGATGCACCCGGTTCTATGGACTCGCATTACTGACCGCCGCCTAAGCCACCCT
CACGTTAAAGTGAACGTGCTTTCTACTTACTACCATCGTTCATTTGAGTTGGCAGACC
ACGGCTACATTTTCAATCCTCAGTCTGACCTTGCAATTGCTAACTTCATCGCAAACTA
CATCATCGAAAACGATGCAGTAAAC

>4637929.3|Sample_4569.R1_contig_29119_1_228_+Vibrio sp. Ex25, 100% identity, napA
TCAGTAAACAAAGGCCTTAACTGTATCAAAGGTTATTTCCTTTCTAAGATCATGTACG
GTGAAGATCGCCTAACTCAACCTCTGCTACGTATGAAAGATGGCAAGTTCCATAAAG
ATGGTGAATTTGCGCCAGTTTCTTGGGACACCGCTTTCGATGTAATGGCAGAGAAGT
GGAAACATGCGCTGAAGAAACAAGGCCCAACCGGTGTAGGTATGTTTGGTTCT

>4637933.3|Sample_4646.R1_contig_20262_1_189_+Vibrio sp. Ex25, 93% identity, napA
TACCGCCTACCAGCGGACATGGTGGTTGCTAACCCTAAACACCGTAAGATTGCAGAA
GACATCTGGAAACTGCCAGAAGGTACGATTCCACCAAAACCTGGCTTCCACGCCGTA
TTGCAAGACCGTATGCTGAATGACGGCGTTCTGAACTGTTACTGGGTTCAATGTAAC
AACAACATGCAAGCC

>4637929.3|Sample_4569.R1_contig_29119_1_228_+Vibrio sp. Ex25, 93% identity, napA
TCAGTAAACAAAGGCCTTAACTGTATCAAAGGTTATTTCCTTTCTAAGATCATGTACG
GTGAAGATCGCCTAACTCAACCTCTGCTACGTATGAAAGATGGCAAGTTCCATAAAG
ATGGTGAATTTGCGCCAGTTTCTTGGGACACCGCTTTCGATGTAATGGCAGAGAAGT
GGAAACATGCGCTGAAGAAACAAGGCCCAACCGGTGTAGGTATGTTTGGTTCT

>4637929.3|Sample_4569.R1_contig_28772_1_191_+Vibrio sp. Ex25, 100% identity, napA
CATGATTTGGCTCCATACGACCGCTACCACCAAGTACGTGGTCTACGTTGGCCTGTTG
TTGATGGTAAGGAAACACAGTGGCGCTTTAAAGAAGGTTCAGACCCATACGCGAAA
GAAGGTTCTGGTTGGGACTTCTACGGCAATGCTGACGGCAAAGCGAAGATCATTTCC
GCGCCATACGAAGCG

>4637933.3|Sample_4646.R1_contig_13573_1_341_+Vibrio splendidus LGP32, 91% identity, napA
GGTACGGGTTGTTCAGTACTGGTAGGTACTCAAAATGGTAAAGTGGTTGCGACTCAA
GGCGACCCAGAAGCACCAGTAAACAAAGGTCTTAACTGTATCAAAGGCTACTTCCTT
ACAAAAATCATGTACGGTAAGGATCGTCTAGATACGCCGCTACTTCGTATGAAAGAT
GGCAAATTCCATAAAGATGGTGATTTCGCACCAGTATCTTGGGATCAAGCTTTCGAC
GTAATGGCTGAAAAGTGGAAAGAAGCGCTGGCTAAGAAAGGTCCAACATCTGTTGG
TATGTTCGGCTCTGGTCAATGGACAGTAATGGAAGGTTATGCTGCTGCAAAAATG

>4637934.3|Sample_4647.R1_contig_5394_1_212_+Vibrio sp. Ex25, 100% identity, napA
TGTATGAACCAGCATACTCGTGGTGTATGGATGAATAACTTGGTTTACAACATCCAC
CTACTAACCGGTAAGATCTCGACTCCGGGGAACAGCCCATTCTCACTAACGGGCCAA
CCATCAGCATGTGGTACCGCTCGTGAGGTTGGTACGTTTGCTCACCGCCTACCAGCG
GACATGGTGGTTGCTAACCCTAAACACCGTAAGATTGCA

>4637933.3|Sample_4646.R1_contig_14221_1_182_+Vibrio splendidus, 90% identity, napA
TCTGGAACACAACATTCCCAAATCGAGGGTGAAGATGAAAATGACAAGAGTGCGTT
TGTGAAAGCAAACGCGGCTGCATCAGCTGCTGCTGTCGCAGGTGTGACACTACCAGC
ATCTGCAGCCAACCTGATTGCAAGCTCTGATCAAACCAAAATCACATGGGACAAAGC
ACTTGTC

>4637930.3|Sample_4570.R1_contig_7739_1_179_+Vibrio sp. Ex25, 100% identity, napA
TGTCTATTTGAGGAATACGCTCAGTTCGGTCGCGGCCATGGCCACGATTTAGCACCG
TATGATCGTTACCACGAAGTACGCGGTTTACGTTGGCCGGTAGTAGATGGTAAAGAA
ACGCTTTGGCGCTACAAAGAAGGCTCAGATCCCTATGCAAAAGCGGGTTCAGGTTGG
GACTTC

>4637930.3|Sample_4570.R1_contig_1326_44_246_+Vibrio sp. Ex25, 100% identity, napA
GCAGAGATGCACCCGGTTCTATGGACTCGCATTACTGACCGCCGCCTAAGCCACCCT
CACGTTAAAGTGAACGTGCTTTCTACTTACTACCATCGTTCATTTGAGTTGGCAGACC
ACGGC
TACATTTTCAATCCTCAGTCTGACCTTGCAATTGCTAACTTCATCGCAAACTACATCA
TCGAAAACGATGCAGTAAAC

>4637930.3|Sample_4570.R1_contig_31537_1_205_+Vibrio sp. Ex25, 93% identity, napA
GTGGGCACAGTCGGTCAAGCAAAATCGGACCTTTGGCAACTAATGGAGTTCTCGAAA
CGCTTCAAAATGGAAGAAGTGTGGCCTGAAGAGCTTCTAGCGAAAGCGCCTCAATAC
CGTGGTAAAACAATGTACGACGTACTTTACCGCAATGGTAACGTCGACAAATTCCCT
ATCGAAGAAGCTCGTGAACTGAATGACGAT

>4637929.3|Sample_4569.R1_contig_23858_1_207_+Vibrio sp. Ex25, 100% identity, napA
CCTATCGAAGAAGCAAATGAATTGAACGATGACGCTCATCACTTTGGCTATTACGTT
CAAAAAGGTCTATTTGAGGAATACGCTCAGTTCGGTCGCGGCCATGGCCACGATTTA
GCACCGTATGATCGTTACCACGAAGTACGCGGTTTACGTTGGCCGGTAGTAGATGGT
AAAGAAACGCTTTGGCGCTACAAAGAAGGCTCA

>4637933.3|Sample_4646.R1_contig_20262_1_189_+Vibrio sp. Ex25, 98% identity, napA
TACCGCCTACCAGCGGACATGGTGGTTGCTAACCCTAAACACCGTAAGATTGCAGAA
GACATCTGGAAACTGCCAGAAGGTACGATTCCACCAAAACCTGGCTTCCACGCCGTA
TTGCAAGACCGTATGCTGAATGACGGCGTTCTGAACTGTTACTGGGTTCAATGTAAC
AACAACATGCAAGCC
