# Supplementary material for: Copepod-Associated Gammaproteobacteria Respire Nitrate in the Open Ocean Surface Layers
Source: Front Microbiol. 2018 Oct 10;9:2390. doi: 10.3389/fmicb.2018.02390 (PMC6194322; doi:10.3389/fmicb.2018.02390)
Supplement: Supplementary file 7 [file Table_5.pdf]

Table S5. Reads from all the samples with metatranscriptomes collected in 2013 that had reads that mapped to *narG* or *narY*, with the identity of the closest match shown as identified by MG-RAST. Sample identifiers are as follows: 4550 - five *Undinula* copepods picked from a net tow in 2013; 4570 - Preliminary Experiment from station SS#13 end time point; 4648 - Preliminary Experiment from station E3 end time point.

>4637935.3|Sample_4648.R1_ contig_17258_1_226_- Marinobacter aquaeolei nitrate reductase, alpha subunit 96%identity, narG

TTCGCGGGCGGCCACGGCGAAACCCACGAAGTCAGTCGCGAGTGGGAAGACAGCTA
CCGCCAGCGCTGGCAGCACGACAAGATCGTGCGTTCCACCCACGGCGTGAACTGCAC
CGGCTCCTGCAGCTGGAAGATTTACGTCAAGAACGGTCTGGTAACCTGGGAAACCCA
GCAAACCGATTACCCCCGTACCCGTCCGGACCTGCCCAACCACGAGCCACAC

>4622665.3|Sample_4550.R1_ contig_2446_1_219_-E. coli str. K-12 substr. W3110, nitrate reductase, beta subunit, 100% identity, narY

CAGATTGATGATTACTACGAACCTTTCACCTTCGACTACGAACATTTGCATAGCGCAC
CGGAAGGCAAACATATTCCTACTGCTCGCCCGCGTTCACTGATTGACGGCAAACGGA
TGGACAAAGTGATCTGGGGGCCAAACTGGGAAGAACTGCTGGGCGGTGAGTTCGAA
AAACGTGCCCGCGACCGCAACTTCGAGGCCATGCAAAAGGAGATA

>4637930.3|Sample_4570.R1_contig_27463_1_301_- Pseudoalteromonas lipolytica, nitrate reductase, alpha subunit 98% identity, narG

GCTGGTCACGGTGTTACCACTGATGAAAATCGCGATTGGGAAGACAGTTATCGCCGC
CGCTGGCAGCACGATAAAATTGTCCGTTCGACTCATGGAGTGAACTGTACCGGTTCT
TGTAGCTGGAAAATTTACGTCAAAGACGGACTTGTTACTTGGGAAACGCAGCAAACC
GATTACCCCGTACTCGCCCAGACTTACCAAACCATGAACCTCGCGGCTGTCCTCGCG
GTGCTAGCTACTCATGGTACATCTATAGTGCAAACCGTCTTAAGCACCCTAAAGTTC
GCCAAGCGCTAT
