# Supplementary material for: Copepod-Associated Gammaproteobacteria Respire Nitrate in the Open Ocean Surface Layers
Source: Front Microbiol. 2018 Oct 10;9:2390. doi: 10.3389/fmicb.2018.02390 (PMC6194322; doi:10.3389/fmicb.2018.02390)
Supplement: Supplementary file 8 [file Data_Sheet_1.docx]

**Copepod-associated Gammaproteobacteria respire nitrate in the open ocean surface layers**

Pia H. Moisander^1^, Katyanne M. Shoemaker^1^, Meaghan C. Daley^1^, Elizabeth McCliment^1^, Jennifer Larkum^2^, Mark A. Altabet^2^

^1^Department of Biology, University of Massachusetts Dartmouth, 285 Old Westport Road, North Dartmouth, MA 02747, USA

^2^School of Marine Science and Technology, University of Massachusetts Dartmouth, 706 South Rodney French Blvd, New Bedford, MA 02744, USA

**Supplementary Materials and Methods**

*Sampling and experimental design: Experiments in 2013*

Preliminary experiments were conducted onboard R/V *Atlantic Explorer* during a cruise to the North Atlantic subtropical gyre in August 1-10, 2013. Copepods (6-17 individuals per vial) were collected and picked to incubations as described for experiments in 2014 (see below and Materials and Methods in the main text). Preliminary experiments investigated N_2_ fixation, denitrification, ammonia oxidation, or nitrite oxidation, each assessed in different experiments. Duplicate treatments were included for a range of substrates and treatments to explore potential metabolisms present. The preliminary rates data from these experiments were used to design experiments in 2014 (due to insufficient replication, rate data from 2013 are not shown). Separate vials were incubated for rate measurements and RNA samples; the latter were collected at the beginning and termination of the experiments and filtered directly on 0.2 μm filters. Additional DNA and RNA samples were saved directly from net tows. Select RNA and DNA samples collected in 2013 were included in samples discussed here (Table S2). To screen for a range of genes potentially expressed in the copepod samples, metatranscriptome libraries were prepared during a cruise conducted in 2013. RNA samples collected at the termination of the experiments, and copepod samples preserved directly from the net tows were included in the metatranscriptome libraries (Tables S2, S3).

*Nutrient and stable isotope analytical methods*

**NH_4_^+^, NO_3_^-^, NO_2_^-^** - Nutrient concentrations of inorganic nitrogen were determined colorimetrically using the SmartChem 200 discrete analyzer (Unity Scientific) and conventional, EPA approved methods. Ammonium analysis was based on the formation of the indophenols-blue complex. Nitrate was converted to nitrite using a Cadmium column and then NO_3_ + NO_2_^-^ are measured after the addition of Greiss reagents (sulfanilamide and NED). Nitrite was measured using the same reagents and this value was subtracted from the combined NO_3_+ NO_2_^-^ measurement to determine nitrate.

**Inorganic Nitrogen Isotope** samples (δ^15^N (‰ versus atmospheric N_2_)) from NR experiments were collected in 125-mL acid washed high density polyethylene bottles and stored frozen until analysis. Azide was used to convert NO_2_^-^ to N_2_O (McIlvin and Altabet 2005) for δ^15^N-NO_2_^-^ analysis on a continuous flow GV IsoPrime IRMS; where δ^15^N_NO2_ = (R_sample_ – R_reference_)/ R_reference_ x 1000) and R is the ratio of masses 15/14. Internal standards (determined by EA-IRMS) were used for calibration for the natural range of ^15^N-NO_2_^-^. To analyze for δ^15^N-NH_4_^+^, a hypobromite solution was used to convert NH_4_^+^ to NO_2_^-^ (Zhang et al. 2007). This was followed by the azide method to convert NO_2_^-^ to N_2_O for isotopic analysis. Internationally recognized standards are analyzed (IAEA N1, USGS25 and USGS26) for calibration purposes during each run. All-N experiments were analyzed for the formation of labeled ^15^N_2_.

**^15^NO_2_^-^** – Azide was used to convert NO_2_^-^ to N_2_O for isotope analysis on a continuous flow IRMS (Zhang et al. 2007). Internal standards (determined by EA-IRMS) were used for calibration for the natural range of ^15^N (McIlvin and Altabet 2005).

**^15^NH_4_^+^** – The NH_4_^+^ was converted to NO_2_^-^ using a hypobromite solution, followed by the azide method to convert NO_2_^-^ to N_2_O for isotopic analysis (Zhang et al. 2007). Internationally recognized standards were analyzed (IAEA N1, USGS25 and USGS26) for calibration purposes during each run.

**^15^NO_3_^-^** – These tracer samples were measured using Vanadium (III) Chloride in a 10% Hydrochloric acid solution with the addition of a 2M sodium azide solution to convert NO_3_^-^ to NO_2_^-^ and then N_2_O (Altabet, unpublished). The method does not measure ^18^O, and is less precise than other methods, but works well for tracer experiments when enrichments are being detected. Internationally recognized standards are analyzed (IAEA N3, USGS34 and USGS35) during each run.

**^15^N-N_2_** – Formation was measured in All-N experiments via continuous flow IRMS. Analyses of δ^15^N_2_ were carried out on a custom made gas extraction system coupled to an IRMS multicollector (GV IsoPrime), as previously described (Charoenpong et al. 2014), with the exception that the IRMS collector cups were set for masses 28, 29, and 30 to capture all N_2_ formed.

**O_2_** – measured by IRMS during the ^15^N_2_ analysis and quantified based on peak height and volume pumped to determine μM present, modifying a previously published method (Charoenpong et al. 2014).

*PCR and RT PCR for amplification of napA and narG*

DNA was extracted on whole individual or pooled zooplankton samples, or on 0.2-μm and 10-μm filters using a modified Qiagen DNeasy Plant Mini kit (Valencia, CA) protocol (Moisander et al. 2008, Shoemaker and Moisander 2015). Before proceeding with the kit, the samples were homogenized (Mini-BeadBeater-8, Biospec Products, Bartlesville, OK) for 2 min at full speed in the lysis buffer, then 45 μL Proteinase K was added (20 mg mL^-1^) (IBI Scientific, Peosta, IA), followed by an incubation for 1 h at 55°C. 4 μL RNase A was added, and tubes incubated at 65°C for 10 min. After the incubation, the samples were vortexed and spun down, and the filters from the water samples were then removed using a sterile needle, while in the case of zooplankton samples picked into tubes without filters, the zooplankton carcasses were left in the tubes. The rest of the extraction steps followed the Qiagen Plant Minikit protocol, with a final elution volume of 50-100 μL. The column purification step was omitted for the experiment 6 samples that were washed by ethanol precipitation, using the wash buffers from the same kit.

RNA samples were extracted using the Qiagen RNeasy minikit (Qiagen). Samples were first agitated in a bead beater three times for 2 min, and cooled on ice between rounds of bead beater treatment. The samples were centrifuged for 2 min at 8,000 g, the filters were removed, the samples were centrifuged again, and supernatant then removed into clean tubes. The rest of the protocol followed the manufacturer's instructions, including an on-column DNase step (1 h in the room temperature). Complementary DNA (cDNA) was made from the RNA separately for *napA* and *narG* using the Superscript III kit (Thermo Fisher), using 1:1 mix of inner and outer reverse *narG* and *napA* primers (see below). A no-RT control was prepared for each RNA sample for one or both of the primer sets. In addition, a no template control sample was prepared for both RT and no-RT master mixes. All of these controls were used as template in parallel PCR reactions with the RT samples. Only few of the no-RT samples showed bands in subsequent PCR reactions and these samples were not used in the downstream steps.

The membrane bound NR (*narG*) and periplasmic NR (*napA*) were amplified using nested PCR protocols for *narG* (Gregory et al. 2000) and *napA* (Flanagan 2000). The *narG* PCR reactions contained 4 μL MgCl (25 mM), 2.5 μL 10X buffer, 2.5 μL primers at 20 μM (T37 and T39 during the first round and W9 and T39 during round two), 0.5 μL dNTPs (10 mM), 0.19 μL Taq DNA polymerase (Thermo Fisher Scientific, EP0402), and 5 μL DNA template (5 μL template from the first round during the second round), adjusted to 25 μL with nuclease free water. The PCR conditions during the first round of PCR were 5 min at 95°C, followed by 37 cycles of 1 min at 94°C, 1 min at 58°C, and 1.5 min at 72°C, then a final 10 min at 72°C and hold at 4°C. During the second round, the PCR conditions were an initial 2 min at 95°C, followed by 37 cycles of 1 min at 94°C, 30 s at 55°C, and 1 min at 72°C, then a final 10 min at 72°C, and hold at 4°C.

The *napA* PCR reactions contained 4 μL (first round) or 3 μL (second round) MgCl (25 mM), 2.5 μL 10X buffer, 0.6 μL primers at 20 μM (V16 and V17 during the first round and V66 and V67 during round two), 0.5 μL dNTPs (10 mM), 0.19 μL Taq polymerase (Fisher), and 5 μL DNA template (5 μL template from the first round during the second round), adjusted to 25 μL with nuclease free water. The PCR conditions during both first and second rounds of PCR were 2 min at 94°C, followed by 37 cycles of 1 min at 94°C, 1 min at 55°C, and 2 min at 72°C, then a final 10 min at 72°C and hold at 4°C. The PCR reactions were run on a BioRad C1000 (Hercules, CA, USA) or the Applied Biosystems 2720 GeneAmp® Thermocycler.

The PCR products were separated on a 1.2% TAE gel electrophoresis, and bands excised and purified using the Thermo Fisher Genejet gel purification kit. The products were ligated into the pGEM-t vector (Promega) and cloned into competent *E. coli* JM109 cells. Plasmid minipreps were purified using the Thermo Fisher Genejet miniprep kit. Sequencing was conducted at the Massachusetts General Hospitals sequencing center (Cambridge, MA, USA) and sequences were trimmed using the CLC Workbench (Cambridge, MA, USA). Databases for *narG* and *napA* were built by downloading sequences from the FunGenes (http://fungene.cme.msu.edu/) repository. The databases were brought to Arb (Ludwig et al. 2004), sequences from this study were imported, and the sequences were conceptually translated in Arb. Protein alignments were created using Clustal W in Mega (Tamura et al. 2011). Additional sequences were imported from NCBI that were close matches to blastn searches for key representatives from different clusters of the sequences recovered in this study. Phylogenetic trees were build using the conceptually translated sequences and bootstrapping for 1000 repetitions included. The trees and bootstrapping were done in Mega and final visualization and annotation done in iTol. The GenBank accession numbers for this study are MH586847-MH586927 for *napA* and MH586928-MH587013 for *narG*.

*Metatranscriptome library preparation and analysis*

The RNA of each sample (extracted as described above from the samples consisting of the >0.2 μm size fraction) was cleaned and concentrated with the addition of 150 μL 100% ethanol, 5 μL glycogen, and 5 μL of 3M NaOAc (per 50 μL extract). The solution was vortexed and stored at -20°C overnight before a 30-min cold centrifugation at maximum speed and 4°C. The supernatant was removed and pellet washed twice more in 350 μL of 70% ethanol. The samples were centrifuged at max speed at 4°C for 10 min for each wash, and the supernatant was discarded. The dried pellet was eluted in 16 μL of RNase-free water. Ribosomal RNA was removed according to the Microb*Express* Kit manufacturer’s protocol (Life Technologies, Beverly, MA). An additional ribosomal RNA removal step was conducted with Terminator Exonuclease enzyme (Epicentre, Madison, WI). For this step, duplicate 20 μL reactions were prepared, each containing 10 μL RNA, 2 μL Terminator Buffer A, 1 μL Terminator Exonuclease, and 7 μL RNAse free water. Reactions were incubated at 30°C for one h in a thermocycler (BioRad C1000, Hercules, CA) with a heated lid, and terminated by the addition of 2 μL of 100 mM EDTA. RNA was precipitated with 2.5 volumes of 70% ethanol and resuspended in 11 μL of RNase-free water. Poly-A tails were added to prokaryotic mRNA with the Epicentre Poly(A) Polymerase Tailing Kit. Reactions were prepared with final concentrations of 1X Poly(A) Polymerase reaction buffer, 1 mM ATP, approximately 1 μg of RNA template, and 1 μL of Poly(A) Polymerase, in RNase-free water at 20 μL total reaction volumes. Reactions were incubated in a thermocycler at 37°C with lid temperature held constant at 50°C for 15-25 min. The reactions were immediately stored at -20°C for at least 15 min to deactivate the enzyme. First and second strand synthesis and cDNA purification of A-tailed mRNA was then performed according to the manufacturer’s protocol with the MessageAmp II Kit (Life Technologies, Beverly, MA, USA). Library preparation of double stranded cDNA for Illumina sequencing was done following the Nextera XT DNA sample preparation guide (Illumina, San Diego, CA). 1 ng of cDNA of each sample was submitted for sequencing on the MiSeq platform (PE250) at Tufts University Core Facility for Genomics (Boston, MA, USA).

Pairing of sequences was done during import to CLC Genomics Workbench 7 (CLC Bio, Aarhus, Denmark). CLC was used to trim reads from the ends based on adaptor and read length. Reads shorter than 64 bp were discarded. Poly-A/T tails were removed with a stand-alone perl script by Jennifer Meneghin (2010): http://alrlab.research.pdx.edu/aquificales/scripts/trim_fasta.pl. Contiguous sequences were assembled using the *de novo* assembly function of CLC Genomics Workbench. The minimum length of contigs used for analysis was 100 bp (Table S3). The short average contig depth reflects the relatively low sequencing depth in the libraries. Contigs were uploaded into the MG-RAST metagenomics analysis server for metatranscriptome annotation (Meyer et al. 2008). Sequences corresponding to Enzyme Commission (EC) number 1.7.99.4 for dissimilatory nitrate reductase were searched in the NCBI database using the tblastx function to find the closest taxonomic identity.

**References**

Charoenpong, C. N., Bristow L. A., and Altabet M. A. (2014). A continuous flow isotope ratio mass spectrometry method for high precision determination of dissolved gas ratios and isotopic composition. *Limnol. Oceanogr. Meth.* **12**, 323-337.

Gregory, L. G., Karakas-Sen A., Richardson D. J., and Spiro S. (2000). Detection of genes for membrane-bound nitrate reductase in nitrate-respiring bacteria and in community DNA. *FEMS Microb. Lett.* **183**, 275-279.

Ludwig, W., Strunk O., Westram R., Richter L., Meier H., Yadhukumar, et al. (2004). ARB: a software environment for sequence data. *Nucleic Ac. Res.* **32**, 1363-1371.

McIlvin, M. R., and Altabet M. A. (2005). Chemical conversion of nitrate and nitrite to nitrous oxide for nitrogen and oxygen isotopic analysis in freshwater and seawater. *Anal. Chem.* **77**, 5589-5595.

Meyer, F., Paarmann D., D'Souza M., Olson R., Glass E. M., Kubal M., et al. (2008). The metagenomics RAST server - a public resource for the automatic phylogenetic and functional analysis of metagenomes. *BMC Bioinformatics* **9**, 386.

Moisander, P. H., Beinart R. A., Voss M., and Zehr J. P. (2008). Diversity and abundance of diazotrophic microorganisms in the South China Sea during intermonsoon. *The ISME J.* **2**, 954-967.

Shoemaker, K. M., and Moisander P. H. (2015). Microbial diversity associated with copepods in the North Atlantic subtropical gyre. *FEMS Microb. Ecol.* **91**, fiv064

Tamura, K., Peterson D., Stecher G., Nei M., and Kumar S. (2011). MEGA5: molecular evolutionary genetics analysis using maximum likelihood, evolutionary distance, and maximum parsimony methods. *Mol Biol Ecol* **10**, 2731-2739.

Zhang, L., Altabet M. A., Wu T., and Hadas O. (2007). Sensitive measurement of NH4+ 15N/14N (delta 15NH4+) at natural abundance levels in fresh and saltwaters. *Anal. Chem.* **79**, 5297-5303.
